# Short-Peptide Supramolecular Hydrogels for In Situ Growth of Metal–Organic Framework-Peptide Biocomposites

**DOI:** 10.1021/acsami.3c06943

**Published:** 2023-06-30

**Authors:** Sara Illescas-Lopez, Javier D. Martin-Romera, Mari C. Mañas-Torres, Modesto T. Lopez-Lopez, Juan M. Cuerva, José A. Gavira, Francisco J. Carmona, Luis Álvarez de Cienfuegos

**Affiliations:** † Departamento de Química Orgánica, Unidad de Excelencia Química Aplicada a Biomedicina y Medioambiente (UEQ), Universidad de Granada, C. U. Fuentenueva, Avda. Severo Ochoa s/n, E-18071 Granada, Spain; ‡ Departamento de Química Inorgánica, UEQ, Universidad de Granada, C. U. Fuentenueva, Avda. Severo Ochoa s/n, E-18071 Granada, Spain; § Departamento de Física Aplicada, Universidad de Granada, C. U. Fuentenueva, Avda. Severo Ochoa s/n, E-18071 Granada, Spain; ∥ Instituto de Investigación Biosanitaria ibs.GRANADA, Av. De Madrid, 15, 18016 Granada, Spain; ⊥ Laboratorio de Estudios Cristalográficos, Instituto Andaluz de Ciencias de la Tierra, Consejo Superior de Investigaciones Científicas-UGR, Avenida de las Palmeras 4, 18100 Armilla, Granada, Spain

**Keywords:** supramolecular hydrogels, metal−organic frameworks, short peptides, biocomposites, composite materials

## Abstract

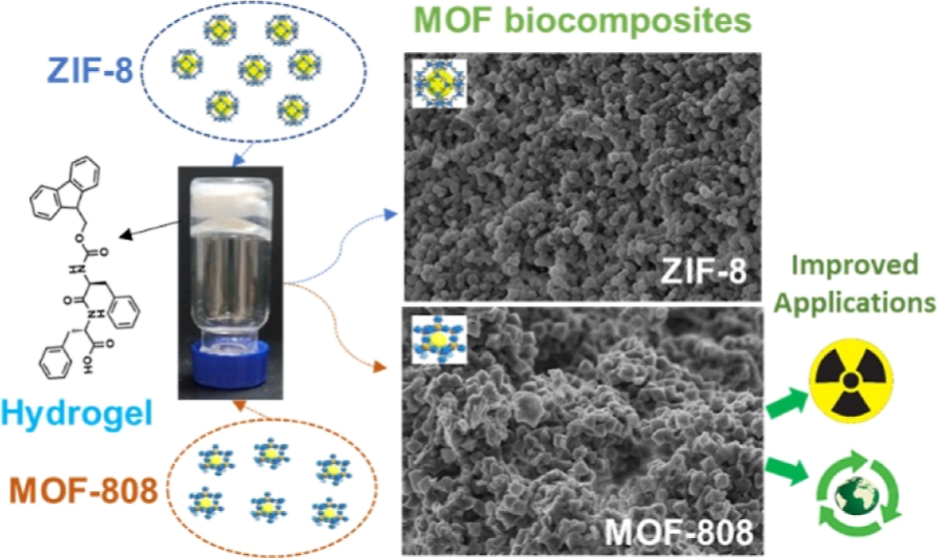

The development of bio-MOFs or MOF biocomposites through the combination of MOFs with biopolymers offers the possibility of expanding the potential applications of MOFs, making use of more environmentally benign processes and reagents and giving rise to a new generation of greener and more bio-oriented composite materials. Now, with the increasing use of MOFs for biotechnological applications, the development of new protocols and materials to obtain novel bio-MOFs compatible with biomedical or biotechnological uses is needed. Herein, and as a proof of concept, we have explored the possibility of using short-peptide supramolecular hydrogels as media to promote the growth of MOF particles, giving rise to a new family of bio-MOFs. Short-peptide supramolecular hydrogels are very versatile materials that have shown excellent in vitro and in vivo biomedical applications such as tissue engineering and drug delivery vehicles, among others. These peptides self-assemble by noncovalent interactions, and, as such, these hydrogels are easily reversible, being more biocompatible and biodegradable. These peptides can self-assemble by a multitude of stimuli, such as changes in pH, temperature, solvent, adding salts, enzymatic activity, and so forth. In this work, we have taken advantage of this ability to promote peptide self-assembly with some of the components required to form MOF particles, giving rise to more homogeneous and well-integrated composite materials. Hydrogel formation has been triggered using Zn^2+^ salts, required to form ZIF-8, and formic acid, required to form MOF-808. Two different protocols for the in situ MOF growth have been developed. Finally, the MOF-808 composite hydrogel has been tested for the decontamination of water polluted with phosphate ions as well as for the catalytic degradation of toxic organophosphate methyl paraoxon in an unbuffered solution.

## 1 Introduction

Metal–organic frameworks (MOFs) are crystalline porous materials characterized by a high surface area, tunable structures, and abundant adsorption sites periodically distributed.^1,2^ These features make MOFs excellent candidates as detoxifying adsorbents/catalysts in environmental or medical applications.^3,4^ However, MOFs are generally produced as crystalline powders which hinder their processing and manufacturing as adsorbents and, therefore, limit their real implementation in everyday life technologies.^5^ A strategy to circumvent the abovementioned challenge is the introduction of (bio)polymers in the synthesis of MOFs, affording a new family of composite materials, denoted as MOF biocomposites, with improved properties such as easy processing or superior biocompatibility, among others.^6−10^ The type of biopolymer and the synthetic protocol used to generate the composite material have a significant impact on the physicochemical properties of the resulting MOF biocomposite, offering a multitude of possibilities for the preparation of novel derivatives having new or improved properties.^10^

Among the different strategies to develop MOF biocomposites, the in situ growth favors a more homogeneous MOF dispersion as well as improves its adhesion to the organic matrix.^10^ Biopolymers such as cellulose, cotton, chitosan, alginate, agarose, and gelatin have been used for the in situ growth of HKUST-1, ZIF-8, -90, -67, and MIL-100, affording composite materials in which the activity of the MOF (catalysis, adsorption, antibacterial, photoemission, etc.) has been improved, thanks to a gain in stability and accessibility.^11−19^ Nevertheless, this in situ strategy has been mainly restrained to natural biopolymers, leaving other biocompatible hydrogels unexplored.

As such, we wondered if short-peptide supramolecular hydrogels could be an ideal medium for the in situ growth of MOFs. These hydrogels have shown many advantages such as economic affordability, precise composition, as well as tunable mechanical, chemical, and biological properties, being used in a multitude of fields.^20−26^ These peptides self-assemble into long fibers by the addition of metallic salts^27−31^ which could promote the in situ MOF growth as well as obtain homogeneous composite materials in which MOF particles could be broadly dispersed.

**Figure 1:**
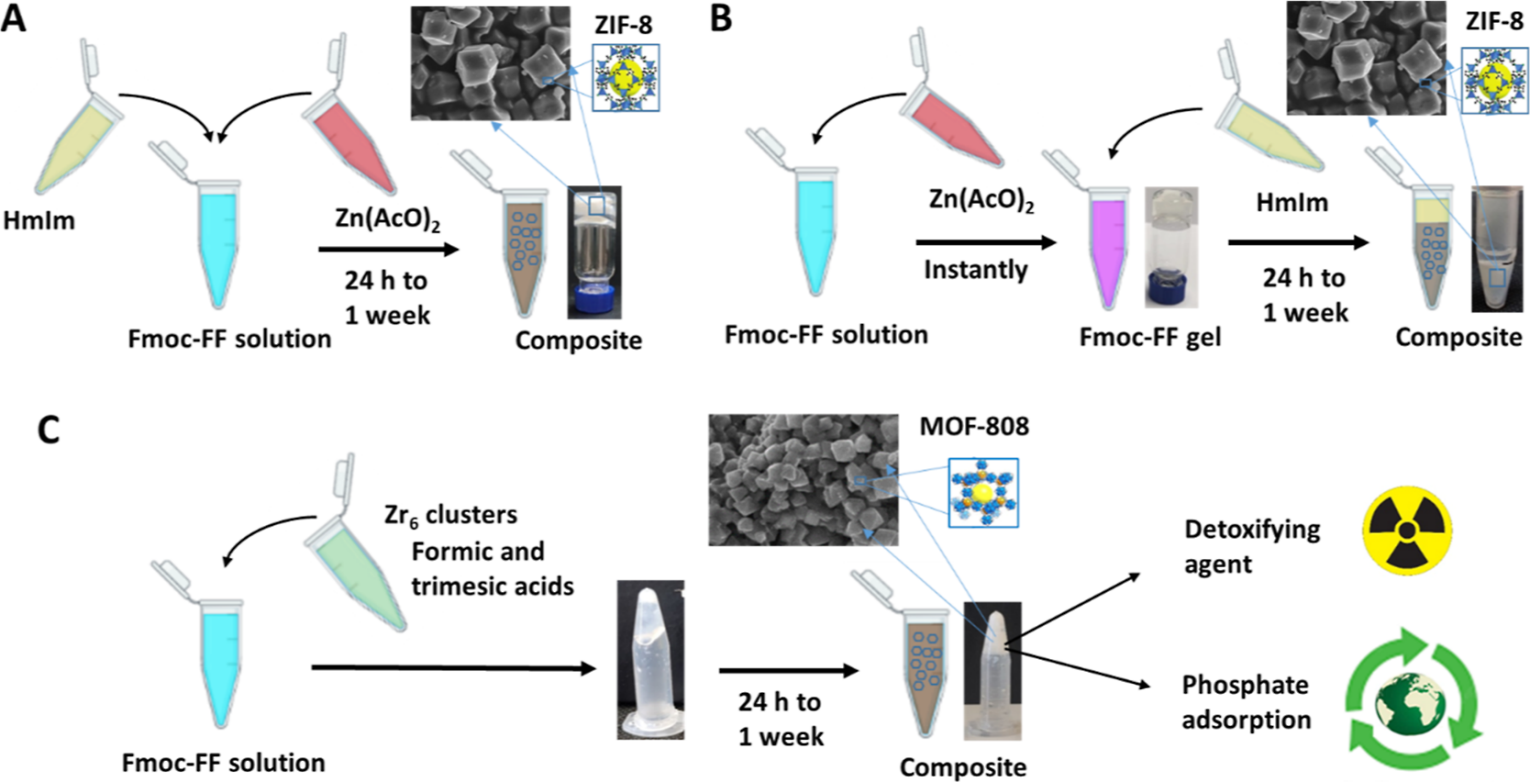
Schematic representation of the different protocols used for MOF synthesis: (A) in situ ZIF-8 growth by simultaneous gel formation (simultaneous protocol); (B) in situ ZIF-8 growth by HmIm diffusion (diffusion protocol); (C) in situ MOF-808 growth by simultaneous gel formation using Zr_6_ clusters as seeds.

In this work, we have studied the conditions required for the in situ growth and preparation of MOF-peptide fiber composites in detail based on two archetypical metal–organic frameworks: ZIF-8 (Zn(mIm)_2_, mIm = 2-methylimidazolate) and MOF-808 (Zr_6_O_4_(OH)_4_(trimesate)_2_(formate)_6_). In the first step, Fmoc-diphenylalanine (Fmoc-FF)^32,33^ and Fmoc-dialanine (Fmoc-AA)^34^ have been used as media to promote the growth ZIF-8. For this purpose, the promotion of peptide self-assembly has been carried out by the addition of Zn(OAc)_2_ acting as seeds for the nucleation of ZIF-8. The amount and ratio of Zn(OAc)_2_ versus 2-methylimidazole (HmIm), the peptide concentration, as well as time and temperature have been screened for optimal conditions. Two different protocols, (1) diffusion protocol, which is in situ MOF growth by diffusion of HmIm over preformed Zn-peptide hydrogels, and (2) simultaneous protocol, which is in situ MOF growth by simultaneous promotion of peptide self-assembly and gelation, have been studied (Figure 1). The influence of the peptide fibers on the size, crystallinity, polymorphism, and morphology of ZIF-8 has been evaluated. Additionally, we have succeeded in obtaining the MOF-808-Fmoc-FF peptide biocomposites following the simultaneous protocol. In this case, the formation of the hydrogel has been achieved by a process of pH switch using a component (formic acid) of MOF-808.

Finally, we have taken advantage of the ability of MOF-808 to effectively detoxify water containing P-based pollutants^35,36^ to evaluate if the integration of the MOF in the peptide hydrogel resulted in novel catalysts/adsorbents with improved properties. To do it, we have compared the capability of the biocomposite and the pristine MOF for detoxifying an aqueous solution containing two P-contaminant models: phosphate ions, typically found in eutrophicated water bodies, and methyl paraoxon, a toxic organophosphorus pesticide (Figure 1).

## 2 Results and Discussion

### 2.1 Gel Formation by the Addition of Zn(OAc)_2_

**Figure 2:**
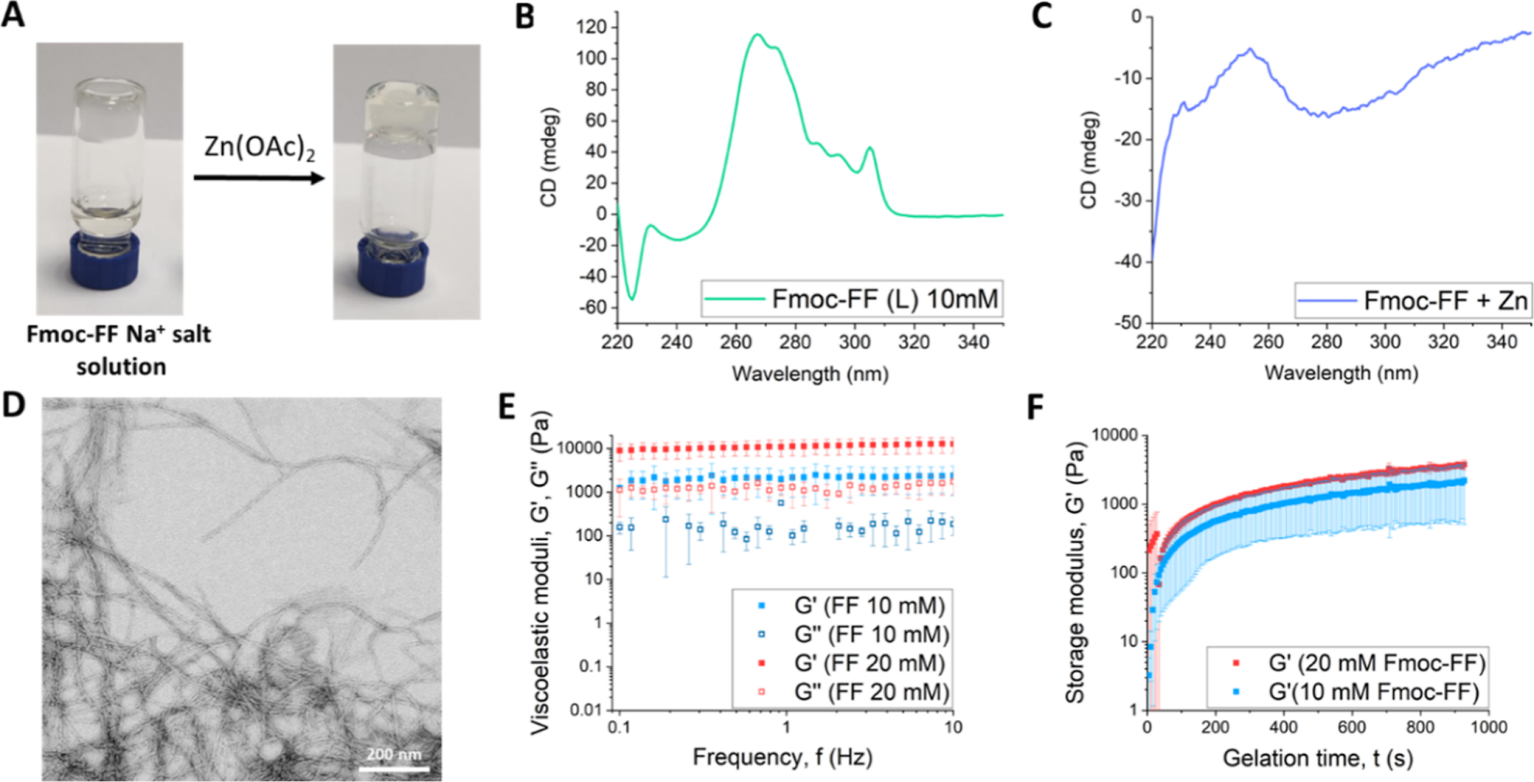
Characterization of Fmoc-FF hydrogels; (A) gel formation by the addition of Zn(OAc)_2_; (B) CD spectra of the Fmoc-FF Na^+^ salt solution (10 mM); (C) CD spectra of the Fmoc-FF hydrogel after Zn(OAc)_2_ addition; (D) TEM image of the Fmoc-FF Zn^2+^ dried hydrogel; (E) mechanical spectra under shear; (F) evolution of the storage modulus (*G*′) during gelation.

We evaluated the capacity to produce ZIF-8 using two different protocols, (1) starting with preformed gels and (2) inducing the simultaneous gel and MOF formation. The success of both methods depended on the ability of Fmoc-dipeptides to form hydrogels after the addition of Zn(OAc)_2_. Thus, we evaluated the self-assembly capacity of Fmoc-FF (10 and 20 mM) after adding a solution of Zn(OAc)_2_ (50 mM) to a pre-existing Fmoc-FF Na^+^ salt aqueous solution (see the Experimental Section). It is known that the self-assembly of Nap-^27−29,37^ and Fmoc-peptides is modulated by metal ions. In the case of Fmoc-peptides, metal ions have shown an influence on the mechanism of growth^38^ and on the secondary structure of the resulting peptide fibrils.^39^ We have recently shown that the nature of the metallic ion also has an influence on the kinetics of the self-assembly process.^31^ Considering this, we could observe that after the addition of the Zn^2+^ salt (50 mM), translucent hydrogels of Fmoc-FF at 10 and 20 mM were formed immediately (Figure 2A). Circular dichroism (CD) spectra of the Fmoc-FF Na^+^ salt solution (10 mM) changed drastically after the addition of Zn^2+^, as reported previously,^39^ giving rise to a preferred β-sheet conformation (Figure 2B,C). Transmission electron microscopy (TEM) of both xerogels (dried hydrogels) confirmed the presence of micron-long fibers with diameters from 10 to 20 nm, very characteristic of this type of peptide (Figure 2D for 10 mM concentrations). To study the influence of the metal cation on the mechanical properties of the hydrogels, rheological measurements were performed (Figure 2E,F). Results of frequency sweeps (mechanical spectra) (Figure 2E) demonstrated a typical gel-like behavior, characterized by values of both the storage (*G*′) and loss (*G*″) moduli almost independent of the frequency of oscillation and with *G*′ being considerably larger than *G*″. This gel-like behavior was clearer for Fmoc-FF 20 mM than for Fmoc-FF 10 mM, something logical considering the larger concentration of peptides in the former than in the latter. A similar trend and values of the viscoelastic moduli were reported in previous work for a Fmoc-FF gel (10 mM) in the presence of Ca^2+^ ions.^31^ In another work, a similar trend although with much lower values of *G*′ and *G*″ was reported for the Fmoc-FF gel in the presence of Zn^2+^, which seems reasonable in view of the much lower peptide concentration (2 mg/mL).^39^ Similarly, during gelation (Figure 2F), *G*′ increased more quickly for Fmoc-FF 20 mM than for Fmoc-FF 10 mM, an expectable behavior as a function of the peptide concentration.

### 2.2 Diffusion Protocol: In Situ ZIF-8 Growth by Diffusion of HmIm over Preformed Gels

**Figure 3:**
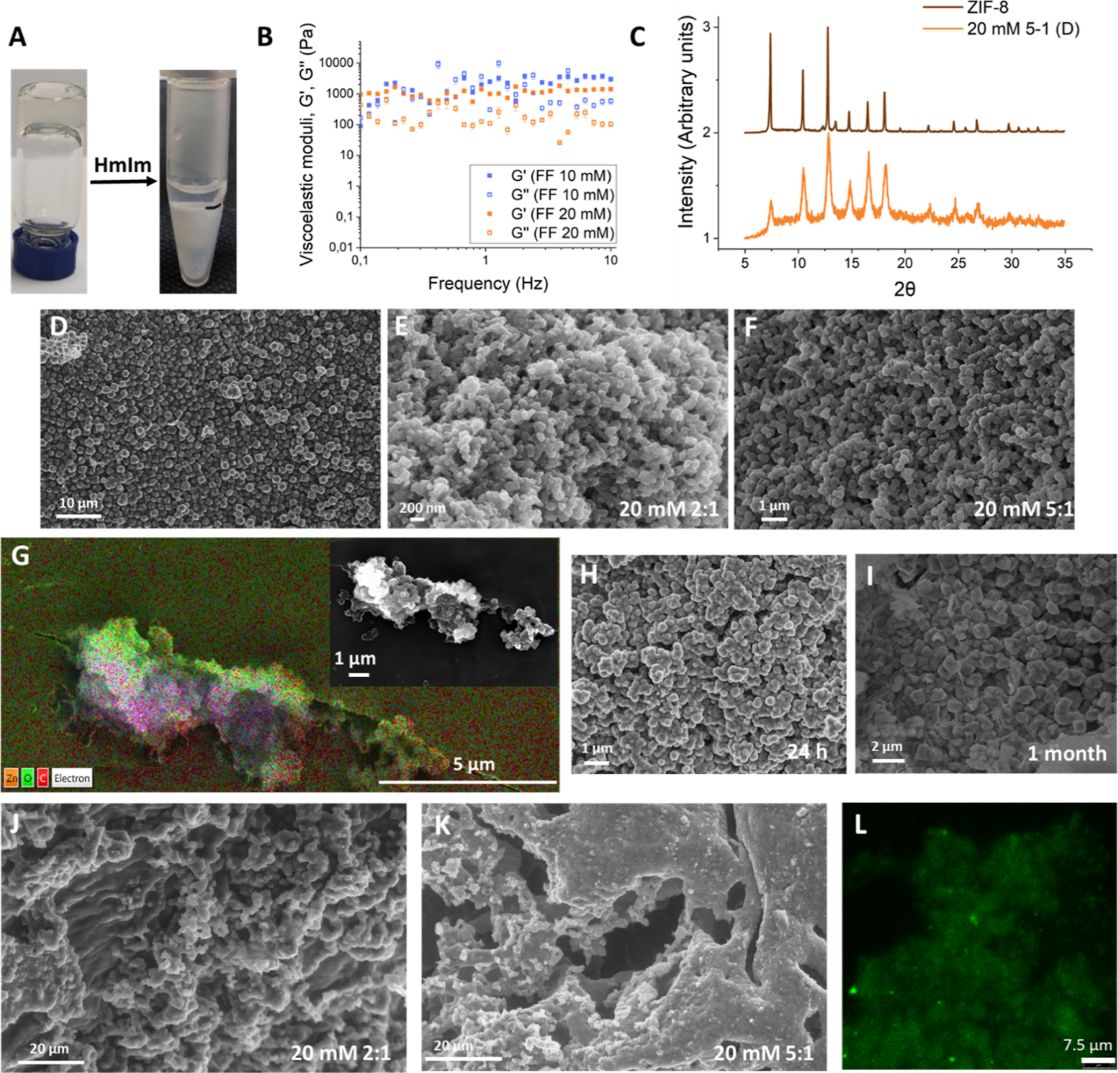
(A) Picture of a composite hydrogel after HmIm diffusion; (B) rheology of composite hydrogels (Fmoc-FF 10 and 20 mM; HmIm/Zn ratio of 5:1) after 1 week of incubation; (C) XRPD pattern of ZIF-8 obtained in water (in brown) and that obtained in the Fmoc-FF Zn^2+^ hydrogel (in orange) at a HmIm/Zn ratio of 5:1 after 1 week of incubation; (D) SEM picture of ZIF-8 obtained in water; (E,F) SEM pictures of ZIF-8 obtained in the Fmoc-FF Zn^2+^ hydrogel after 1 week of incubation at a HmIm/Zn ratio of 2:1 and 5:1, respectively; (G) EDX analysis of the ZIF-8 composite (Fmoc-FF 10 mM; HmIm/Zn ratio of 5:1) after 1 week of incubation; (H,I) SEM picture of ZIF-8 obtained in the Fmoc-FF Zn^2+^ hydrogel after 24 h and 1 month of incubation, respectively; (J,K) ESEM pictures of ZIF-8—Fmoc-FF Zn^2+^ composite hydrogels after 1 week of incubation at a HmIm/Zn ratio of 2:1 and 5:1, respectively; (L) CLSM of the ZIF-8 composite hydrogel (Fmoc-FF 20 mM, HmIm/Zn ratio of 5:1) after 1 week of incubation.

Once the gel nature of Fmoc-FF Zn^2+^ salts was confirmed, the first MOF synthetic protocol was tested. Thus, a solution of HmIm was added on top of the hydrogel (10 and 20 mM). Different HmIm concentrations of 100, 250, and 500 mM were tested, with the ratio of HmIm/Zn being 2, 5, and 10, respectively. The HmIm solution was allowed to diffuse for 24 h in the hydrogel. The diffusion of HmIm through the hydrogel phase gave rise to the appearance of a white precipitate. After 24 h, the whole hydrogel volume appeared white (Figure 3A). After 1 week of incubation, the mechanical properties of composite hydrogels (HmIm/Zn ratio of 5:1) were evaluated. The values of viscoelastic moduli as a function of frequency (mechanical spectra) (Figure 3B) revealed a strong weakening with respect to hydrogels without diffusion of HmIm, especially for 20 mM Fmoc-FF (*G*′ of the order of 1,000 after diffusion of HmIm vs 10,000 for hydrogels without HmIm). A similar conclusion is inferred from the curves of *G*′ and *G*″ vs shear stress amplitude (Figure S1). Furthermore, while in the absence of HmIm, the trends of *G*′ and *G*″ vs frequency (Figure 2E) revealed a typical gel behavior (i.e., moduli almost independent of frequency and *G*′/*G*″ ≈ 10), samples after diffusion of HmIm demonstrated erratic trends for both *G*′ and *G*″ vs frequency, with *G*′/*G*″ < 10, which are more typical of not well-formed gels. From these results, a degradation of the gel structure after diffusion of HmIm can be concluded. This degradation should be likely due to the requirement of Zn for the growth of the MOF, competing with the Fmoc-FF fibers that also required Zn for their growth and for maintaining their integrity. Next, composite hydrogels were freeze-dried and analyzed. X-ray powder diffraction (XRPD) analysis showed that, for all the HmIm/Zn ratios tested (2, 5, and 10), ZIF-8 was formed as an exclusive crystalline polymorph, although at a ratio of 2:1, some impurities appeared (Figure 3C for a 5:1 ratio). This result is very relevant considering the same protocol but in an aqueous solution and keeping the same pH values and variations (see the Experimental Section) was only able to afford ZIF-8 at a HmIm/Zn ratio of 10:1. At a ratio of 5:1, another nonporous polymorph, dia(Zn),^40^ was exclusively formed and at a ratio of 2:1, an amorphous material was formed (Figure S2). Particles obtained in water showed micron sizes (1.2 ± 0.2 μm on average) and rhombic dodecahedron shape Figure 3D for a scanning electron microscopy (SEM) picture. Other reported protocols, in water^41−43^ and in the gel phase,^19^ have shown that the ratio HmIm/Zn required to form ZIF-8 has to be higher than 10:1. The reason why a much smaller proportion of imidazole is required to form ZIF-8 in this system is not clear, but preliminary results suggest that Zn cations are well dispersed in the peptide fibers being readily accessible to interact with imidazole molecules, acting as nucleation centers for the formation of ZIF-8 crystals. In fact, SEM images of the white precipitate phase showed the presence of a multitude of significantly small (0.14 ± 0.02 to 0.25 ± 0.06 μm) ZIF-8 crystals well distributed over all the observed area, showing a crystallization process in which nucleation was extremely enhanced even at a HmIm/Zn ratio of 2:1 (Figure 3E,F). Energy-dispersive X-ray spectroscopy (EDX) analysis of ZIF-8 composites (Fmoc-FF 10 mM; HmIm/Zn ratio of 5:1) showed a uniform distribution of Zn within the composites (Figures 3G and S3).

The morphology of the particles also suggested a fast nucleation process, presenting shapes between cubic and spherical with no clearly defined edges. Nevertheless, the morphological analysis of these samples was restrained due to the presence of the peptide fibers covering the particles. Similar sizes and morphologies have been described for ZIF-8 particles formed in water when the ratio of HmIm/Zn increases to 35, 70, and 100.^41−43^ In this case, the higher amount of HmIm promotes the nucleation, leading to the formation of many particles of smaller sizes. The formation of cubic and spherical particles presenting a high-energy surface versus the low-energy surface of a rhombic dodecahedron with truncated corners, which is the equilibrium form of ZIF-8 crystals, shows again that these particles have formed from a high supersaturation of nuclei presented in the media.^44^ Due to the presence of peptide fibers, the morphological evolution of these particles is significantly restrained. SEM images of samples after 24 h of incubation showed particles of the same size but more spherical in shape (Figure 3H). Samples incubated at 37 °C for 1 month showed better-defined faces and higher sizes, showing that, like what happens in water,^41^ these crystals can evolve over time (Figure 3I).

To get a better picture of the composite material, environmental SEM (ESEM) of the hydrogel samples was also performed. This technique allows the preservation of the 3D hydrogel structure avoiding its collapse, and therefore, it is very useful to study the morphology of the hydrogel and, as in this case, how well integrated the different components of the hydrogel are. Figure 3J,K shows the ESEM images of composite hydrogels obtained at HmIm/Zn ratios of 2:1 and 5:1. The hydrogel structure appeared completely covered by MOF particles, showing the good integration of both materials (peptide fibers and MOF). The homogeneous distribution of the MOF particles and their homogeneous and small sizes suggest that peptide fibers act as nucleation centers through their Zn^2+^ chelation. We have observed a similar process in the biomineralization of hydroxyapatite but in this case mediated by Ca^2+^.^30^ Composite hydrogels (Fmoc-FF 20 mM; HmIm/Zn ratio of 5:1) were also observed by confocal laser scanning microscopy (CLSM) as Fmoc-peptides and ZIF-8 are fluorescent. Figure 3L shows a uniform basal green fluorescence emission from peptide fibers decorated with tiny spherical particles corresponding to ZIF-8 composite particles (see Figure S4 for CLSM of ZIF-8 grown in water and Fmoc-FF hydrogels). This analysis shows again the homogeneous distribution of ZIF-8 particles throughout the peptide network.

Next, we evaluated the influence of the peptide nature, concentration, and temperature on the MOF crystallization process. Hydrogels of Fmoc-FF at 10 mM and Fmoc-AA at 10 and 20 mM were tested using a ratio HmIm/Zn of 2:1 and incubating the sample for a week at 37 and at 4 °C. ZIF-8 crystals of similar sizes and purity were obtained with both peptide hydrogels, concentrations, and different temperatures, although MOF particles seemed to be more crystalline at 37 °C (Figure S5). Scale-up processes at prefixed conditions of Fmoc-FF and Fmoc-AA at 20 mM, a ratio of HmIm/Zn of 2:1, and at 37 °C were also tested using a reaction volume increased by 10-fold. Results show that, for both peptides, ZIF-8 was obtained with the same crystallinity as the preliminary tests (Figure S6).

ZIF-8 composite hydrogels (Fmoc-FF 20 mM; HmIm/Zn ratio of 5:1) were allowed to dry at 37 °C for 48 h. Dried samples were observed by optical microscopy (×2 zoom) showing a film-like smooth surface in which amorphous MOF aggregates were not observed (Figure S7). Dried samples were slowly rehydrated by the subsequent addition of small amounts of water (5 μL). ZIF-8 composites were hydrophilic and had the capacity to absorb water (15 μL), showing a swelling ratio of 1.6%. The subsequent addition of water resulted in the release of the material into the surrounding water.

### 2.3 Simultaneous Protocol: In Situ ZIF-8 Growth by Simultaneous Gel Formation

**Figure 4:**
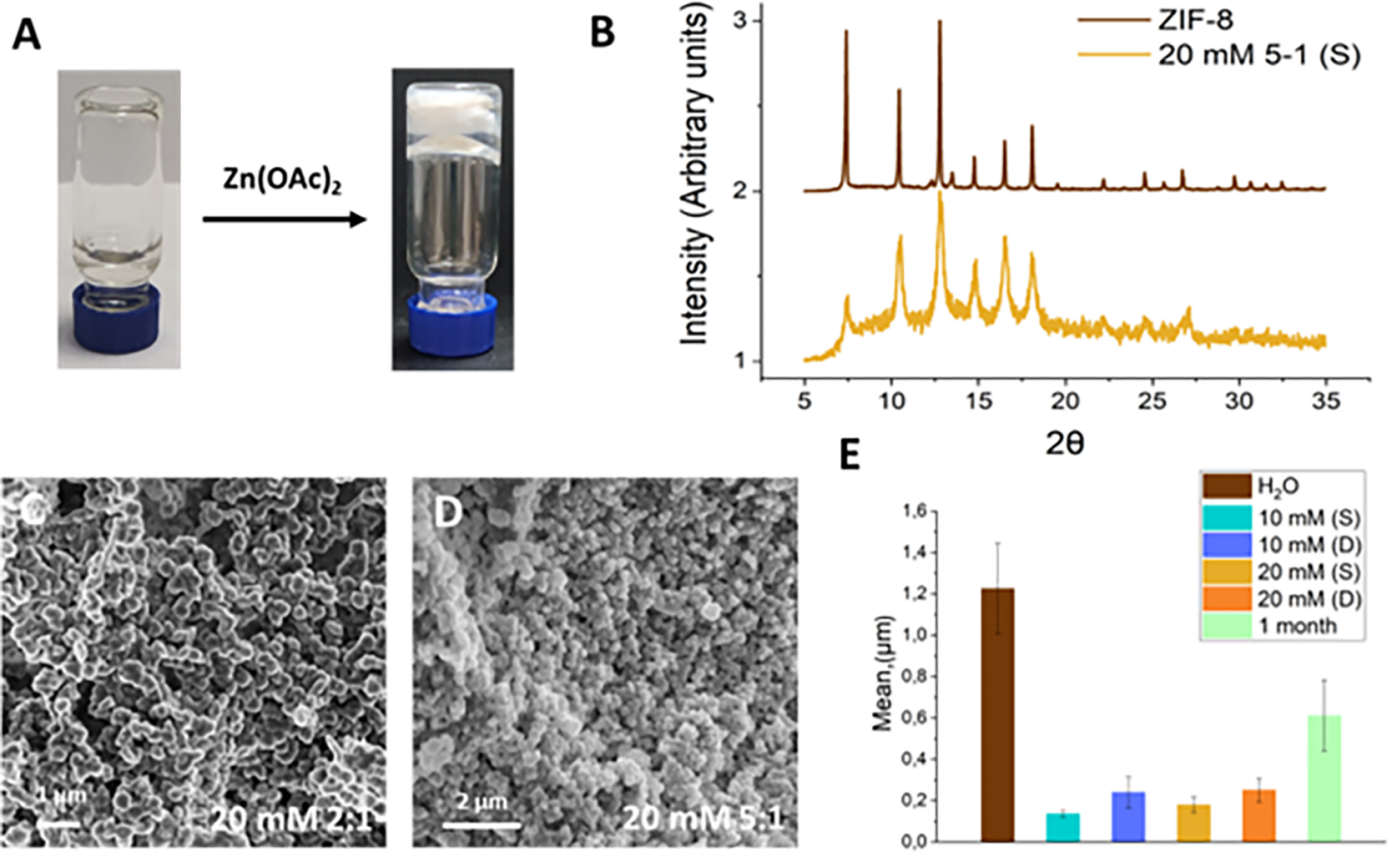
(A) Picture of the composite hydrogel after Zn(OAc)_2_ addition; (B) XRPD pattern of ZIF-8 obtained in water (in brown) and that obtained in the Fmoc-FF Zn^2+^ hydrogel (in mustard) at a HmIm/Zn ratio of 5:1; (C,D) ZIF-8 obtained in the Fmoc-FF Zn^2+^ hydrogel after 1 week of incubation at HmIm/Zn ratios of 2:1 and 5:1, respectively; (E) Mean size of ZIF-8 particles obtained in water and in both protocols (S means simultaneous; D means diffusion).

First, we tried to promote the formation of the gel and MOF particles by mixing a peptide basic solution (Fmoc-FF 10 mM and 20 mM) with HmIm and Zn(OAc)_2_ (50 mM). Same as in the diffusion protocol, three different HmIm/Zn ratios of (2, 5, and 10) were tested. After mixing the three components, translucent gels formed instantaneously for all the different HmIm concentrations tested (Figure 4A). Equal to the diffusion protocol, the mechanical properties of the composite hydrogels were considerably affected after the formation of the MOF (Figure S1). XRPD analysis at the two peptide concentrations showed the formation of ZIF-8 as an exclusive crystalline polymorph, in high purity at 10 and 5 HmIm/Zn ratios (even better using Fmoc-FF at 20 mM) (Figure 4B) and with some impurities at a 2:1 ratio. SEM images of the samples at HmIm/Zn ratios of 2:1 and 5:1 showed ZIF-8 particles of similar shape and size to those obtained with the diffusion protocol (Figure 4C,D). EDX and CLSM analysis of the composites also showed similar results to those obtained with the diffusion protocol (Figures S8 and S9). ZIF-8 particles grown in Fmoc-FF at 10 and 20 mM at an HmIm/Zn ratio of 5:1 were measured, and size histograms were plotted (see the Experimental Section). Figure 4E shows the mean size of the particles obtained by both protocols. Although the mean size is almost the same for both protocols and peptide concentrations, particles of slightly bigger sizes were obtained by the diffusion protocol which is not surprising considering that, in this protocol, a diffusion gradient that can influence particle size occurs, similar to what we have observed for protein crystallization in these hydrogels.^45,46^

Additional tests were performed in which the amount of HmIm was even lower, at ratios of HmIm/Zn of 1:1 and 0.5:1, but in this case, ZIF-8 was not formed. Finally, the amount of Zn was also reduced to 20 mM and 10 mM. At a ratio of HmIm/Zn of 5:1 (Zn^2+^ 20 mM), SEM images showed the presence of small aggregates composed of stacked thin layers like those reported by Jian et al.^41^ and ascribed to an intermediate phase in the synthesis of ZIF-8. At a Zn^2+^ concentration of 10 mM, these aggregates were hard to identify (Figure S10).

Noteworthily, ZIF-8 is one of the most studied MOFs for reversibly hosting drugs.^47−49^ In this regard, porosity is an important aspect to be considered in the application of MOF-based composites as drug delivery systems. Therefore, we finally assessed the porosity of ZIF-8 hydrogel composites. Specifically, we selected the composites formed by means of the diffusion protocol (HmIm/Zn ratio of 5:1) with Fmoc-FF at 10 and 20 mM. N_2_ adsorption analysis indicates that both dried composites (xerogels) are microporous, proving the permanent porosity of ZIF-8 particles grown in the gel after thermal activation (Figure S11). N_2_ uptake at 77 K for the different materials inversely correlates with their content of peptide (BET surface area of 1625, 1090, and 775 m^2^ g^–1^ for ZIF-8 microparticles and ZIF-8 composites of Fmoc-FF at 10 and 20 mM, respectively) due to the nonmicroporous nature of the Fmoc-FF hydrogel.

### 2.4 Protocol 2. In Situ MOF-808 Growth by Simultaneous Gel Formation

Next, we evaluate the capacity of Fmoc-FF peptides to form other types of MOFs. We focused our attention on MOF-808 since a recent protocol reports its synthesis at room temperature and in water, starting from preformed Zr_6_ oxoclusters.^50^ Recently, we have also developed a green synthesis using water-based microwave heating.^36^ This MOF is particularly interesting for its stability and promising detoxification activity of water contaminated with phosphorus-based compounds, like phosphate ions or organophosphate (OP) pesticides.^51^ In fact, we have shown that this MOF is able to capture relevant amounts of phosphate ions from water while simultaneously degrading a toxic organophosphorus pesticide, methyl paraoxon.^36^ Organophosphate toxicity relies on the capability of OP compounds to strongly bind to acetylcholinesterase (AChE), the enzyme that regulates neurotransmission, and inhibits its enzymatic activity. The consequences of AChE inhibition are paralysis, respiratory failures, seizures, or even death. In this regard, MOF-808 has also proved to act as a potential antidote for the treatment of OP-poisoning by removing a toxic OP-compound from simulated physiological media and, therefore, avoiding the interaction of the toxic molecule with the targeted AChE enzyme.^52^

**Figure 5:**
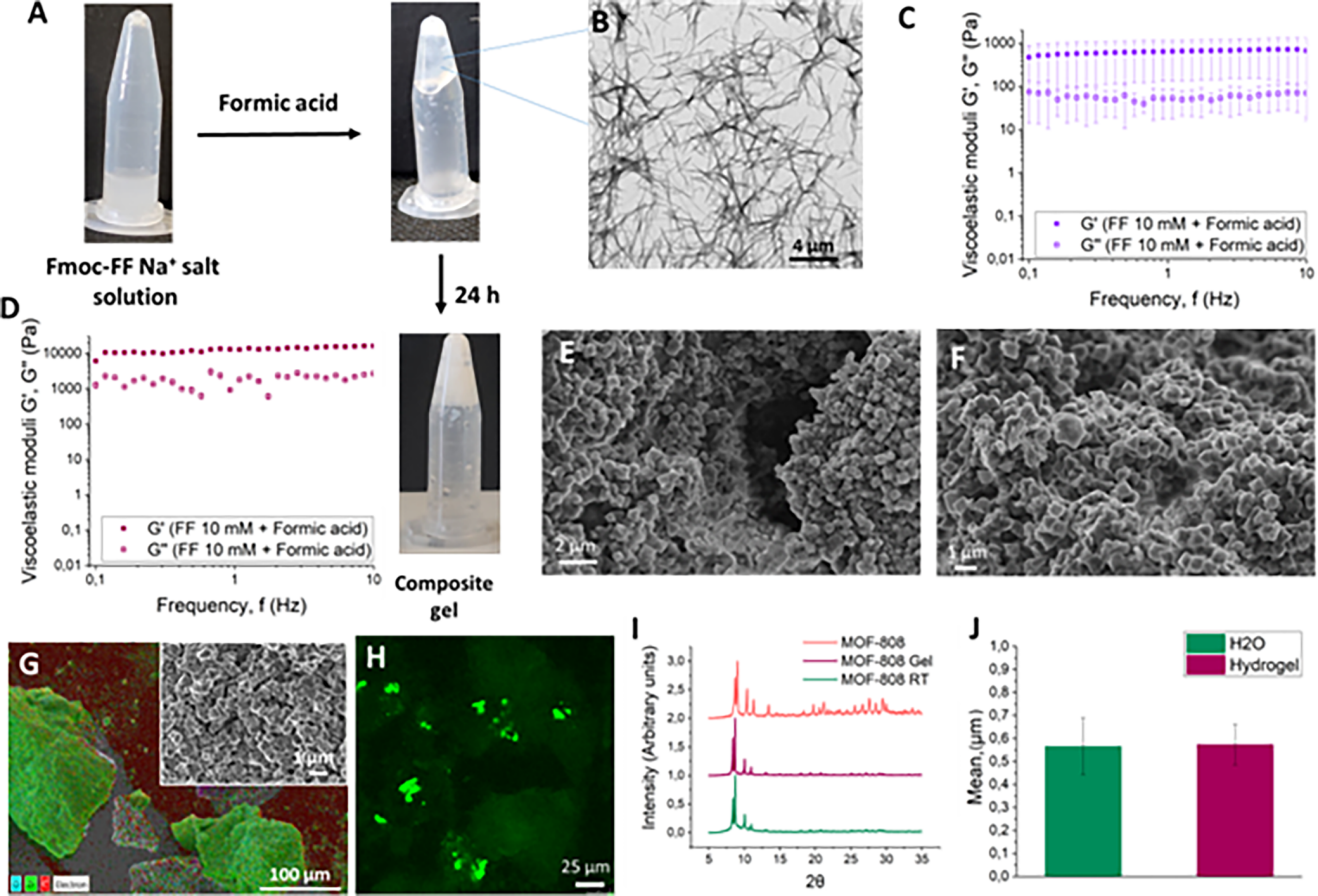
(A) Picture of the gel after addition of formic acid and composite-MOF-808 formation after 24 h; (B) TEM picture of Fmoc-FF formed by the addition of formic acid; (C) mechanical spectra under shear of the hydrogel (Fmoc-FF 10 mM); (D) mechanical spectra of the composite hydrogel (Fmoc-FF 10 mM) after 1 week of incubation; (E) SEM picture of MOF-808 obtained in water; (F) SEM picture of MOF-808 obtained in the Fmoc-FF hydrogel after 1 week of incubation; (G) EDX analysis of the MOF-808 composite (Fmoc-FF 10 mM) after 1 week of incubation; (H) CLSM of the MOF-808 composite hydrogel (Fmoc-FF 10 mM) after 1 week of incubation; (I) XRPD pattern of MOF-808 obtained by solvothermal synthesis (in orange), that obtained in the Fmoc-FF hydrogel (in burgundy), and that obtained in water (in green); (J) mean size of MOF-808 particles obtained in water and in the Fmoc-FF hydrogel.

We were able to in situ-grow MOF-808 in the Fmoc-FF hydrogel (10 mM) by simultaneous gel formation, but in this case, gel formation was triggered in the presence of Zr_6_ oxoclusters by lowering the pH by adding formic acid, a component required to form MOF-808 in water (Figure 5A). The addition of formic acid induced the formation of a homogeneous gel instantaneously, showing long peptide fibers as observed by TEM, characteristic of these types of hydrogels (Figure 5B). The mechanical properties of the hydrogel were tested by frequency sweep rheology (mechanical spectra) (Figure 5C). The results demonstrated a gel-like behavior, although of a weaker nature (smaller values of *G*′ and *G*″) than for the equivalent gel prepared by addition of a solution of Zn(OAc)_2_ (50 mM) to the pre-existing Fmoc-FF Na^+^ salt aqueous solution (see Figure 2E). However, gelation was in this case (with formic acid) over 1 order of magnitude faster than for gels prepared by addition of Zn^2+^ (compare Figure 2F with S12). This faster gelation is due to the rapid drop in pH induced by the addition of formic acid. The formation of a white precipitate after 24 h indicated the formation of the MOF (Figure 5A). Rheology of the composite hydrogels showed a strong enhancement of the robustness of the samples with respect to those not containing the MOF (compare Figure 5D with 5C and Supporting Information Figure S12). This result is contradictory to that observed for samples prepared by addition of Zn(OAc)_2_, but it is not surprising. For samples prepared by addition of Zn, the formation of the MOF implied a competition for Zn with the Fmoc-FF fibers. However, in the case of addition of formic acid, Fmoc-FF fibers were built by a change in pH and, consequently, the growth of the MOF did not provoke the degradation of the Fmoc-FF fibers. Instead, the MOF adhered to the Fmoc-FF fibers, making them more robust, which justifies the strengthening manifested by the higher values of *G*′ in the presence of the MOF.

After a period of incubation of 1 week, samples were lyophilized and analyzed by XRPD and SEM. Similar to the results obtained for ZIF-8, SEM pictures showed the presence of a multitude of bipyramidal tetragonal MOF-808 particles very homogeneous in size and similar to those obtained in water (Figure 5E,F). EDX analysis confirmed the presence of the Zr metal distributed throughout the composite material (Figures 5G and S13). CLSM of the composites showed images similar to those obtained for ZIF-8 composites in which MOF particles appeared well integrated within the peptide network (Figures 5H and S14). XRPD analysis of the samples obtained in the gel and in water was practically identical, showing the diffraction pattern described for MOF-808 (Figure 5I). Contrary to the results obtained for the synthesis of ZIF-8, the gel did not show any influence over the size of the MOFs (Figure 5J). In this case, peptide fibers did not compete for any of the MOF components and the gel only acted as a physical medium in which the Zr_6_ seeds were homogeneously distributed. Thanks to the high porosity of these gels,^53^ MOF growth inside them was not restrained, reaching similar sizes to that in water.

Dried samples showed, in this case, irregular surfaces formed by a multitude of spherical aggregates. These samples looked more compacted than those of ZIF-8 and, after addition of water, they did not have the capacity to absorb water, being more hydrophobic (Figure S15).

The combination of two or more components to afford composite materials has been a very useful strategy to develop materials with improved properties.^54,55^ The resulting composites can retain the properties of the individual components but can also give rise to novel properties or present synergistic effects. This implies that composite materials can perform multiple tasks or present multiple functionalities. Biocompatible composite hydrogels have been developed for multiple medical or biotechnological applications, improving stability, biocompatibility, degradability, and so forth.^56^ In this particular case, the possibility of having a biocompatible peptide hydrogel functionalized with MOF-808 known for its adsorption/degradation capabilities affords a biocomposite hydrogel with potentially useful properties that can be applied as novel detoxification or purification platforms.^57^ As such, composite hydrogels were tested as adsorbents toward the detoxification of water containing phosphate ions, a typical P-pollutant with severe negative consequences in aquatic ecosystems like eutrophication. Additionally, we have also evaluated the ability of the composite hydrogel to degrade an OP compound, namely, methyl paraoxon, as a toxic model molecule.

**Figure 6:**
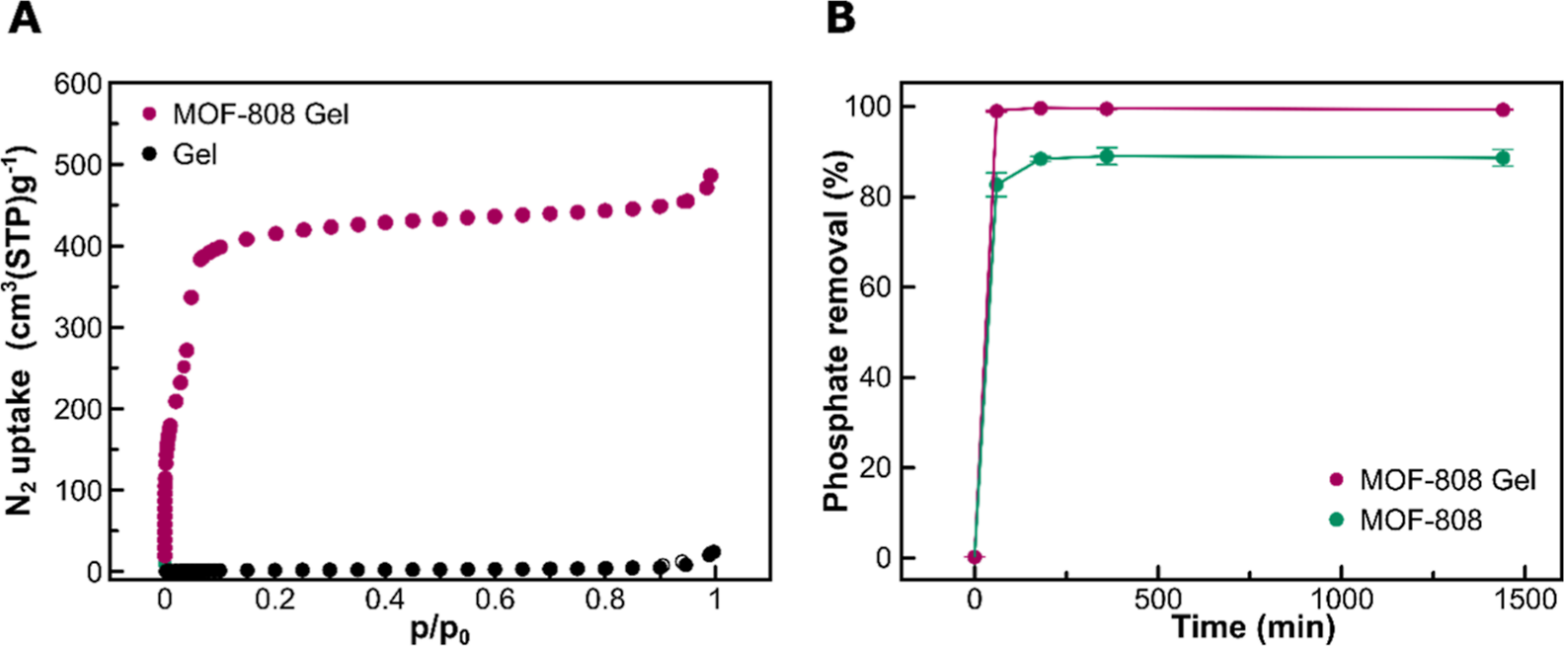
(A) N_2_ adsorption isotherm at 77 K of the dried MOF-808 gel composite (pink circles) and dried Fmoc-FF-gel (black circles); (B) cumulative phosphate removal kinetics by the dried MOF-808 gel composite (pink circles) and MOF-808 particles prepared in water (green circles) at 25 °C.

First, we determined the porosity of MOF-808 particles grown in the gel by gas adsorption. The N_2_ adsorption isotherm at 77 K of the thermally activated dry biocomposite (xerogel) proved its permanent porosity (Figure 6A) with a calculated BET surface area of 1580 m^2^ g^–1^. This value is slightly lower than the one observed for the pristine MOF-808 (2050 m^2^ g^–1^),^36^ probably due to the nonmicroporous nature of the gel component in the biocomposite (S_BET_ = 6.5 m^2^ g^–1^) (Figure 6A). Notwithstanding this, these results suggest that the cavities of the MOF-808 grown in the gel can be accessible to guest molecules like phosphate ions, and therefore, the P-pollutants could be trapped inside the pores. To test this, a phosphate solution (0.41 mM) was added on top of a MOF-808-gel composite in situ grown for 1 week. As a control, a Fmoc-FF hydrogel was also prepared, and it was mixed with an aqueous phosphate solution (0.41 mM). The mixtures were maintained at room temperature, and the evolution of the phosphate concentration was spectrophotometrically evaluated by means of the molybdenum blue method.^58^ As it is shown in Figure S16, the Fmoc-FF hydrogel gel shows a negligible phosphate adsorption capacity after 24 h of incubation due to its lack of phosphate-sorption sites. By contrast, the MOF-gel biocomposite can capture phosphate ions from water, with a cumulative removal of 33.0 ± 1.7 and 55.3 ± 1.6% after 1 and 24 h, respectively. These pieces of evidence certify that the guest molecules (phosphate ions) can diffuse through the gel-MOF biocomposite and reach the sorption sites in the MOF-808 cavities where they are trapped.

Afterward, we compared the phosphate adsorption performance of MOF-808 crystals prepared in water with the adsorption performance shown by the biocomposite. In this case, the dry biocomposite (xerogel) instead of the hydrogel biocomposite was used to avoid diffusion constraints. Specifically, an aqueous solution with a phosphate concentration typically found in wastewater^59^ (0.08 mM) was mixed with MOF-808 crystals prepared in water (109.1 mg L^–1^, 0.080 μmol MOF-808) and with the dried MOF-808 biocomposite (109.1 mg L^–1^ containing 0.024 μmol MOF-808). The resulting suspensions were stirred at 25 °C, and the phosphate concentration in the solution was spectrophotometrically monitored by means of the molybdenum blue method. As it is shown in Figure 6B, both materials show an efficient capture of phosphate ions from water after 24 h, with a cumulative phosphate removal rate of 88 ± 2 and 99.0 ± 0.1% for MOF-808 crystals and the biocomposite, respectively. The MOF-808-Fmoc-FF biocomposite proved to have faster phosphate adsorption kinetics than MOF-808 crystals, reaching the maximum adsorption rate after 60 min of stirring. The growth of MOF-808 particles along the peptide fibers in the biocomposite could favor their exposition to the solution and enhance the diffusion of phosphate ions inside their cavities. Noteworthily, the phosphate concentration after the biocomposite treatment (0.80 ± 0.08 μM) was below the EPA and EU criterion for phosphate content in water (1 μM),^59^ proving the good performance of the new material toward the decontamination of phosphate-polluted water.

On the other hand, several Zr-MOFs have proved to degrade organophosphate compounds in basic buffered media containing organic amines like *N*-ethylmorpholine quickly and efficiently. However, the catalytic activity of these materials in unbuffered media (like pure water) decreases significantly.^60^ Unfortunately, the use of nucleophilic bases like *N*-ethylmorpholine is prevented in certain applications, like wastewater purification or medical treatment for OP poisoning. This fact makes the development of alternative strategies to catalyze the degradation of OP compounds in unbuffered media necessary.

**Figure 7:**
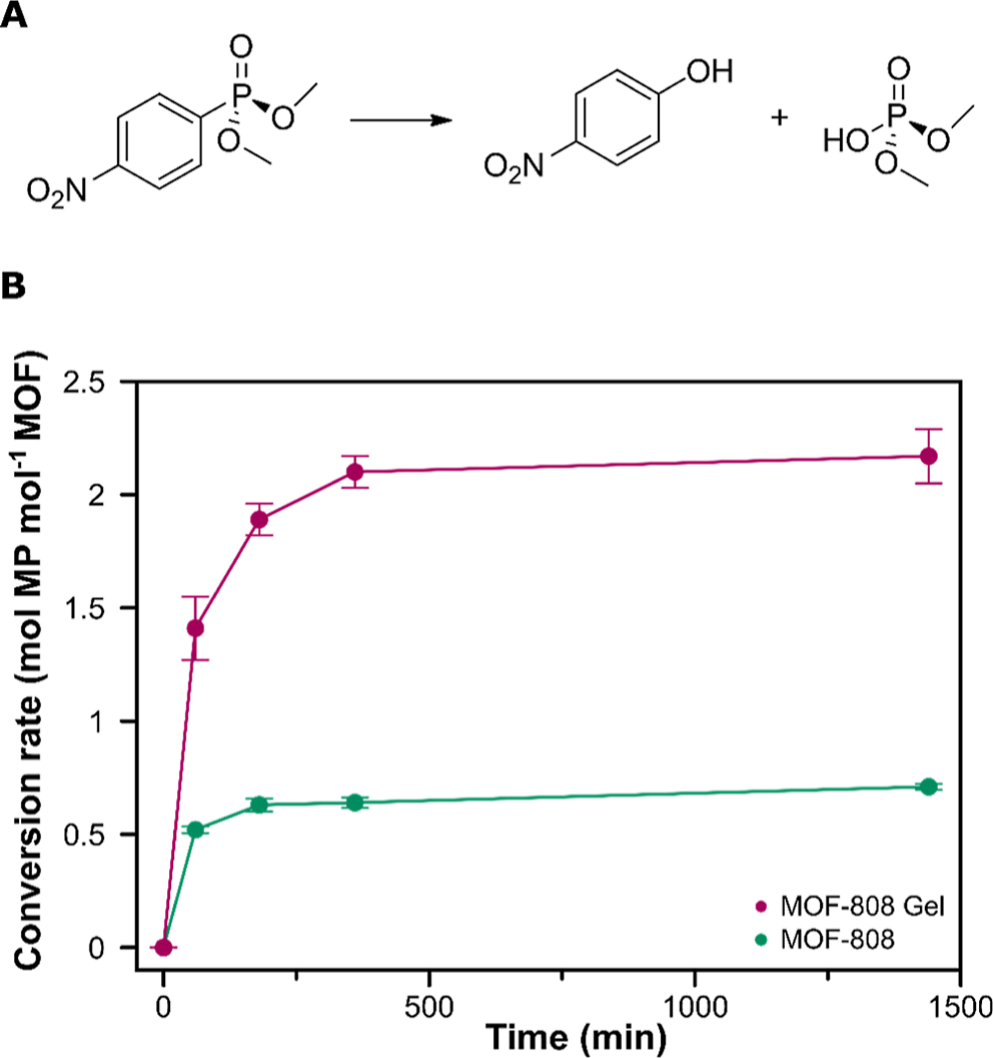
(A) Hydrolysis reaction of methyl paraoxon into 4-nitrophenol and dimethylphosphate. (B) Cumulative hydrolysis of methyl paraoxon catalyzed by the dried MOF-808 gel composite (pink circles) and MOF-808 particles prepared in water (green circles) at 25 °C.

With this aim, we evaluated the ability of the different materials prepared, namely, MOF-808 and Fmoc-FF hydrogel and MOF-808-hydrogel biocomposites, to catalyze the hydrolysis of a toxic organophosphate model (methyl paraoxon, MP, oral rat LD_50_ = 3 mg kg^–1^) into less toxic 4-nitrophenol (oral rat LD_50_ = 667 mg kg^–1^) and dimethylphosphate (oral rat LD_50_ = 3283 mg kg^–1^) in unbuffered aqueous solutions. To test this, MOF-808 (0.22 mg, 0.16 μmol MOF-808), the dry Fmoc-FF xerogel (0.22 mg), and the dry MOF-808-xerogel composite (0.22 mg, 0.05 μmol MOF-808) were exposed to an aqueous solution of methyl paraoxon (0.16 mM, 1 mL) and the mixtures were stirred at room temperature. The progress of the catalytic reaction was followed by quantifying the amount of 4-nitrophenol formed over time by UV–vis spectroscopy. Negligible formation of 4-nitrophenol was observed when the Fmoc-FF xerogel was used as the catalyst (Figure S17). By contrast, MOF-808 crystals and the MOF-808-xerogel composite hydrolyze MP into 4-nitrophenol (Figure 7a). In the latter cases, Lewis-acidic Zr-sites in MOF-808 particles activate the phosphate center in MP, while the nucleophilic OH-sites of the MOF cluster hydrolyze the toxic compound.^60^ Notwithstanding this, while MOF-808 crystals show a moderate MP degradation with a conversion rate of 0.7 mol of MP per mol of MOF after 24 h, the integration of MOF-808 particles in the Fmoc-FF leads to a faster and more efficient degradation performance, with a conversion rate of 1.4 and 2.2 mol of MP per mol of MOF after 60 min and 24 h, respectively (Figure 7b). As previously observed in the phosphate adsorption experiments, the synergistic effect observed in the catalysis driven by the composite is attributed to the homogeneous distribution of MOF-808 particles grown along the peptide fibrils, which enhances the interaction of the catalytic sites on the MOF particle surface with toxic molecules in the solution.

## 3 Conclusions

To sum up, herein, we have explored the use of short-peptide supramolecular hydrogels as media to grow MOF particles to obtain novel MOF biocomposites. The versatility of the hydrogels has allowed us to study different protocols for the synthesis of these composites. As such, we have developed a diffusion protocol in which one of the constituents of the MOF is inside the hydrogel and the other is allowed to diffuse through the gel and a simultaneous protocol in which all the constituents of the MOF are added together in a peptide solution triggering simultaneous gel formation. We have observed that, for the synthesis of ZIF-8, both processes enhance the formation of MOF particles due to the great accessibility of Zn^2+^ cations that electrostatically interact with the peptides. This allows the use of a much lower proportion of HmIm than similar protocols in water, making these processes more efficient and atom-economical. The hydrogel-based protocol leads to the formation of smaller, spherical ZIF-8 particles that extensively decorate the hydrogel peptide network, providing a more homogeneous and well-integrated inorganic–organic composite material. Additionally, these peptides have also been tested for the growth of MOF-808, triggering gel formation with formic acid and using Zr_6_O_4_(OH)_4_(AcO)_12_ seeds as nucleation agents. This protocol also gives rise to the formation of MOF-808 particles with similar characteristics and properties to those obtained in water, highlighting the versatility of these supramolecular hydrogels as media for MOF production. Finally, we have evaluated the benefits of integrating MOF-808 particles in the Fmoc-FF fibrils toward two different applications: phosphate adsorption and catalytic degradation of toxic organophosphates. The distribution of MOF crystals along the peptide fibrils in the biocomposite has proved to favor (i) the diffusion of phosphate ions toward the cavities of the porous matrices, leading to faster phosphate adsorption than in pristine MOF-808 crystals and (ii) the interaction of the catalytic sites on the MOF particle surface with the substrate molecules in the solution, resulting in a 3-fold catalytic activity of MP degradation in comparison with pristine MOF-808 crystals (2.2 and 0.7 mol of MP converted per mol of MOF-808 after 24 h, respectively)

## 4 Experimental Section

### 4.1 Reagents and Materials

*N*-Fluorenylmethoxycarbonyl-diphenylalanine (Fmoc-FF) and *N*-fluorenylmethoxycarbonyl-dialanine (Fmoc-AA) were purchased from LifeTein, USA, and were used without further purification. Zinc acetate (Zn(AcO)_2_)·2 H_2_O, 2-methylimidazole (HmIm), zirconium (IV) tetrachloride (ZrCl_4_), trimesic acid, and methyl paraoxon were purchased from Sigma-Aldrich. Formic acid (98%) was purchased from Acros Organics.

### 4.2 Preparation of Basic Solutions of Peptides

Fmoc-FF and Fmoc-AA peptides were weighed separately into a vial, and deionized water was added to obtain final concentrations of 20 and 30 mM as stock solutions. The suspension was sonicated (in an HSt Powersonic 405-ultrasonic bath) for at least 2 h. Then, a NaOH solution (0.5 M) was added dropwise until a clear solution (pH = 10.7) was obtained. The pH was measured using a HACH Sension pH 3 pH meter. The pH meter was calibrated using pH 4, pH 7, and pH 10 buffer solutions.

### 4.3 In Situ ZIF-8 Growth by Diffusion of HmIm

Gelation of the peptide’s basic solution was induced by adding an aqueous solution of Zn(OAc)_2_ (300 mM) to obtain a Zn^2+^ final concentration of 50 mM. Gelation was completed instantaneously, determined by test tube inversion. After 30 min, an aqueous solution of HmIm was added on top of the Zn hydrogel, allowing it to diffuse for 24 h. Different ratios of HmIm/Zn were evaluated, and 2:1 and 5:1 ratios were selected, in which the amount of HmIm was 100 mM for 2:1 and 250 mM for 5:1. After adding Zn(AcO)_2_, the pH of the solution was 7.1 and 6.9 after the formation of ZIF-8. These pH values were equal to those obtained in the formation of ZIF-8 in water. The system was kept at 37 °C for 1 week to reach equilibrium, as described in water, although after 24 h, a white precipitate appeared.

### 4.4 In Situ ZIF-8 Growth by Simultaneous Promotion of Peptide Self-Assembly

The peptide solution (20 or 30 mM initial peptide concentration) was mixed with an aqueous solution of HmIm (1,8 M) at pH = 10.7 to obtain a mixture with a final peptide concentration of 10 or 20 mM and 100 mM (2:1 ratio) or 250 mM (5:1) mM of HmIm, respectively. Then, an aqueous solution of Zn(OAc)_2_ (300 mM) was added to trigger gel formation (a Zn^2+^ final concentration of 50 mM). Different ratios of HmIm/Zn were evaluated, and 2:1 and 5:1 ratios were selected. The pH after adding the Zn(AcO)_2_ solution was 7.1, equal to the pH obtained in the formation of ZIF-8 in water. The system was kept at 37 °C for 1 week to reach equilibrium, although a white precipitate appeared after 24 h.

### 4.5 Formation of Zr_6_ Oxoclusters

The synthesis of Zr_6_O_4_(OH)_4_(AcO)_12_ clusters was carried out following a described protocol.^50^ In brief, 1 g of zirconium tetrachloride (ZrCl_4_), 1.67 mL of acetic acid, and 2.67 mL of 2-propanol were heated in a Schlenk flask at 120 °C for 1 h. The white precipitate was washed with 2-propanol and recovered by filtration (see Figure S18 for XRPD of the Zr_6_ oxocluster).

### 4.6 In Situ MOF-808 Growth by Simultaneous Promotion of Peptide Self-Assembly

For a final volume of 500 μL, 37.5 mg of Zr_6_O_4_(OH)_4_(AcO)_12_ clusters was added. Formic acid (10 mM final concentration) was added to the clusters and mixed by vortexing. To this solution, water was added and mixed until a completely clear solution was obtained. Finally, trimesic acid (0.03 mM) was added to this solution and mixed. To obtain the gel, a basic solution of Fmoc-FF (for 10 mM final concentration) was added and mixed quickly, giving rise to a hydrogel after 15 min. The system was kept at 37 °C for 1 week until equilibrium was reached, although a white precipitate appeared after 24 h.

In order to quantify the amount of MOF-808 in the MOF-gel biocomposite, 20 mg of the lyophilized MOF-808-gel composite was digested in 600 μL of NaOD 1 M for 24 h at 25 °C. Afterward, 3 μL of *N*,*N*-dimethylacetamide (DMA, final concentration 0.029 M) was added as an internal standard and the concentration of trimesate ions in the solution was determined by ^1^H NMR spectroscopy in a 400 MHz Bruker Nanobay AVANCE III HD spectrometer (CIC, University of Granada) (Figure S19). The results indicate that the MOF-808-gel biocomposite contains 0.22 mmol per gram of material (dry basis).

### 4.7 Phosphate Adsorption Experiments

In the case of the phosphate adsorption experiments performed with the materials in the gel state, 100 μL of the MOF-808-gel biocomposite (10 mM of Fmoc-FF) and 100 μL of Fmoc-FF (10 mM of Fmoc-FF) were formed during 1 week, following the protocol described above. 900 μL of an aqueous solution of K_2_HPO_4_ (0.41 mM) was added on the top of the gel, and the mixtures were kept under orbital shaking at 25 °C for 1, 3, 6, and 24 h. At each time, 100 μL of the supernatant was collected by centrifugation (9168*g*/15 min) and diluted with 900 μL of water. The phosphate concentration in the solutions was determined spectrophotometrically by means of the molybdenum blue method,^58^ using the absorbance maximum at λ = 880 nm. The UV–vis spectra were collected on a Shimadzu UV spectrophotometer. All the experiments were performed in triplicate.

For the phosphate adsorption experiments performed with the dried samples, aqueous solutions of K_2_HPO_4_ (0.08 mM, 1 mL) were mixed with 0.11 mg of the dry gel (10 mM Fmoc-FF), 0.11 mg of dry MOF-808-gel biocomposite (10 mM Fmoc-FF, corresponding to 0.024 μmol MOF-808), or 0.11 mg of MOF-808 (0.080 μmol). The suspensions were shaken at 25 °C for 1, 3, 6, and 24 h. At each time, 800 μL of the supernatant was collected by centrifugation (9168*g*/15 min). The concentration of phosphate ions was determined spectrophotometrically by means of the molybdenum blue method as explained above. All the experiments were performed in triplicate.

### 4.8 Methyl paraoxon Degradation Studies

Aqueous solutions of methyl paraoxon (0.16 mM) were mixed with 0.22 mg of the dry gel (10 mM Fmoc-FF), 0.22 mg of the MOF-808-gel biocomposite (containing 0.05 μmol MOF-808), and 0.22 mg of MOF-808 (0.16 μmol). The suspensions were shaken at 25 °C for 1, 3, 6, and 24 h. Each time, the supernatant was collected by centrifugation (9168*g*/15 min) and analyzed by means of a high-performance liquid chromatography instrument equipped with a DAD detector, Thermo Fisher Scientific SpectraSystem UV-8000, column: silica-based Hypersil GOLD C18 (100 × 4.6 mm I.D., 5 μm particle size, mobile phases: water (A) and acetonitrile (B), gradient: 5–95% B over 15 min, flow rate: 0.8 mL min^–1^, volume of injection: 100 μL). The methyl paraoxon concentration in the supernatants was determined spectrophotometrically (λmax = 271 nm). All the experiments were performed in triplicate.

### 4.9 MOF Particle Size Analysis

Particle size distribution was measured from SEM images using ImageJ 1.47 software. Data were processed with Excel to calculate mean values and standard deviation. Results were expressed as the average size of 100 crystals per sample. In each analyzed image, all crystals with at least one defined face were measured taking the diagonal length of the biggest exposed face.

### 4.10 Rheological Characterization of Hydrogels

Mechanical properties were characterized under oscillatory shear stress using a Bohlin CS10 controlled-stress rheometer (UK) provided with a plate–plate geometry 40 mm in diameter. We subjected the samples to frequency sweep tests of fixed shear stress (τ_0_ = 1 Pa) within the linear viscoelastic region (LVR) and increasing frequency in the range of 0.1–10 Hz. From these measurements, we obtained the storage (*G*′) and loss (*G*″) moduli of the samples as a function of frequency within the LVR (mechanical spectra). Three different samples were measured to ensure the statistical significance of the results. The mean values and standard deviations of each magnitude were provided in this work. Gelation kinetics were investigated by oscillatory shear under fixed stress (τ_0_ = 1 Pa) and frequency (1 Hz), starting from pregel mixtures immediately after their preparation, by using a Bohlin CS10 controlled-stress rheometer (UK) provided with concentric cylinders geometry 14.1 mm in diameter. In these experiments, *G*′ and *G*″ were monitored for 1 h. Three different samples were measured to ensure the statistical significance of the results. The mean values and standard deviations of each magnitude are provided in this work.

### 4.11 Rheological Characterization of Composites

Mechanical properties were characterized under oscillatory shear stress using a Bohlin CS10 controlled-stress rheometer (UK) provided with a plate–plate geometry 40 mm in diameter. We subjected the samples to frequency sweep tests of fixed shear stress (τ_0_ = 0.1 Pa) within the LVR and increasing frequency in the range of 0.1–10 Hz. From these measurements, we obtained the storage (*G*′) and loss (*G*″) moduli of the samples as a function of frequency within the LVR (mechanical spectra). Three different samples were measured to ensure the statistical significance of the results. The mean values and standard errors of each magnitude are provided in this work.

### 4.12 Transmission Electron Microscopy

Dried gels (xerogels) were studied using a LIBRA 120 PLUS Carl Zeiss. Hydrogels were vortexed and diluted twice with water. A drop of the fiber suspension obtained was placed on a 300-mesh copper grid and stained with a uranyl acetate negative stain. The sample was dried at room temperature for 1 h.

### 4.13 Circular Dichroism

Peptide basic solutions and hydrogels were recorded using a spectrophotometer J-815 of Jasco with a xenon lamp of 150 W. The mixtures were jellified into a 0.1 mm quartz cell (Hellma 0.1 mm quartz SuprasilR). Spectra were obtained from 220 to 350 nm with a 1 nm step and 1 s integration time per step at 25 °C.

### 4.14 Scanning Electron Microscopy

Samples were deposited on SEM supports and then were coated with a fine carbon layer and examined by SEM using HITACHI, S-510 equipment.

### 4.15 High-Resolution SEM

Samples were deposited on SEM supports and then were coated with a fine carbon layer and examined by SEM using Carl Zeiss SMT, AURIGA (FIB-FESEM), equipment to acquire EDX data.

### 4.16 Environmental SEM

Refrigerated samples of peptide hydrogels were examined by ESEM using an FEI Quanta 400 equipped with a Peltier effect cooling stage.

### 4.17 X-ray Powder Diffraction

XRPD data were collected on a Bruker D2 PHASER Bruker diffractometer: Cu Kα radiation (λ = 1.5418 Å), measurement range 2θ = 5–35°, time per step = 1.0, and step size = 0.02°. Prior to each measurement, the samples were manually ground in an agate mortar and then deposited in the hollow of a zero-background silicon sample holder.

### 4.18 Nitrogen Adsorption Experiments

The nitrogen adsorption isotherms were measured at 77 K on Micromeritics Tristar 3000 and 3-flex volumetric instruments.

In the case of the MOF-808-based materials, the samples were heated for 8 h at 333 K and outgassed to 10^–1^ Pa before the adsorption measurements.

In the case of the ZIF-8-based materials, namely, ZIF-8 nanoparticles, Fmoc-FF gel, and ZIF-8 composite of Fmoc-FF 10 mM and ZIF-8 composite of Fmoc-FF 20 mM, the materials were soaked in MeOH (15 mL) for 3 days, replacing the solvent with fresh MeOH every 24 h. Afterward, the materials were heated for 12 h under dynamic vacuum (10^–1^ Pa) before the adsorption measurements.

### 4.19 Optical Microscopy

Optical images were recorded using the Image-Focus-Alpha software of the Nikon AZ100 microscope with a zoom of 1 × 1 × 0.6 for MOF-808 and a zoom of 1 × 2 × 0.6 for ZIF-8. The time lapse between each drop of water (5 μL) was 2 min for both MOFs. The final photo was taken after 15 h for MOF-808 and after 1 h for ZIF-8 due to its decay.

### 4.20 Confocal Laser Scanning Microscopy

Supramolecular peptide hydrogels and MOF biocomposites were studied using an Inverted Microscope Leica DMI6000 at a fixed excitation wavelength of 405 nm and an emission read range of 510–545 nm. Samples were observed in their hydrated state.

### 4.21 Swelling Ratio Calculations

The sample of dried ZIF-8 was weighted in a high-precision balance before starting the swelling experiment and after the addition of 15 μL of water. The calculation of the swelling ratio (SR) was made using the following formula
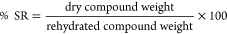


This study was supported by grants PID2020-118498GB-I00 and PID2020-113608RB-I00 funded by MCIN/AEI/10.13039/501100011033, projects P18-FR-3533 and A-FQM-340-UGR20 by FEDER/Junta de Andalucía-Consejería de Transformación Económica, Industria, Conocimiento y Universidades (Spain) and Project PPJIA2021.20 by Universidad de Granada. F.J.C. is thankful for the financial support provided by the Marie Skłodowska-Curie Individual Fellowship (H2020-MSCA-IF-2019-EF-ST-888972-PSustMOF) within the European Union H2020 programme and EU FEDER. M.C.M.-T. acknowledges grant PRE2018-083773 funded by MCIN/AEI/10.13039/501100011033 and by “ESF Investing in your future”, Spain.

## Supporting Information Available

Mechanical properties of Fmoc-FF hydrogels obtained by addition of Zn and ZIF-8 composites obtained by the two different protocols; XRPD of ZIF-8 obtained in water and in the gel at 10:1 and 2:1 HmIm/Zn ratios; EDX analysis of the ZIF-8 composite obtained by the diffusion protocol; CLSM analysis of the ZIF-8 composite obtained by the diffusion protocol compared with ZIF-8 in water and with the Fmoc-FF hydrogel; SEM images and XRPD of ZIF-8 composites obtained with different peptides and temperatures; XRPD of ZIF-8 comparing different peptides and scale-up; rehydration of ZIF-8; EDX analysis of the ZIF-8 composite obtained by the simultaneous protocol; CLSM analysis of the ZIF-8 composite obtained by the simultaneous protocol; SEM images of ZIF-8 obtained by the diffusion protocol with less amount of Zn; N_2_ adsorption isotherms of ZIF-8 and ZIF-8-gel composites; evolution of the storage modulus (*G*′) during gelation of hydrogels prepared by protocol 2 and evolution of viscoelastic moduli of Fmoc-hydrogels formed with formic acid and MOF-808 composite; EDX analysis of the MOF-808 composite; CLSM of the MOF-808 composite compared with the Fmoc-FF hydrogel formed with formic acid and MOF-808 obtained in water; rehydration of MOF-808; phosphate adsorption kinetics of the MOF-808-hydrogel composite and Fmoc-FF hydrogel at room temperature; MP degradation kinetics of dried Fmoc-FF gel at room temperature; XRPD of the Zr_6_ oxocluster; and ^1^H NMR spectrum of the MOF-808-gel composite digested with NaOD 1M at room temperature (Internal Reference: DMA) (PDF)
