# Supplementary material for: Short-Peptide Supramolecular Hydrogels for In Situ Growth of Metal–Organic Framework-Peptide Biocomposites
Source: ACS Appl Mater Interfaces. 2023 Jun 30;15(27):32597–609. doi: 10.1021/acsami.3c06943 (PMC10347120; doi:10.1021/acsami.3c06943)
Supplement: Supplementary file 1 — am3c06943_si_001.pdf [file am3c06943_si_001.pdf]

Supporting Information

Short-peptide supramolecular hydrogels for *in situ* growth of Metal-Organic Framework-Peptide biocomposites.

Sara Illescas-Lopez,[a,#] Javier D. Martin-Romera,[b,#] Mari C. Mañas-Torres,[a] Modesto T. Lopez-Lopez,[c,d] Juan M. Cuerva,[a] José A. Gavira,[e] Francisco J. Carmona,[b]* Luis Álvarez de Cienfuegos[a,d]*

[a] Universidad de Granada (UGR), Departamento de Química Orgánica, Unidad de Excelencia Química Aplicada a Biomedicina y Medioambiente (UEQ), C. U. Fuentenueva, Avda. Severo Ochoa s/n, E-18071 Granada, Spain.

[b] UGR, Departamento de Química Inorgánica, UEQ, C. U. Fuentenueva, Avda. Severo Ochoa s/n, E-18071 Granada.

[c] UGR, Departamento de Física Aplicada, C. U. Fuentenueva, Avda. Severo Ochoa s/n, E-18071 Granada.

[d] Instituto de Investigación Biosanitaria ibs.GRANADA Av. De Madrid, 15, 18016, Granada, Spain.

[e] Laboratorio de Estudios Cristalográficos, Instituto Andaluz de Ciencias de la Tierra, Consejo Superior de Investigaciones Científicas-UGR, Avenida de las Palmeras 4, 18100 Armilla, Granada, Spain.

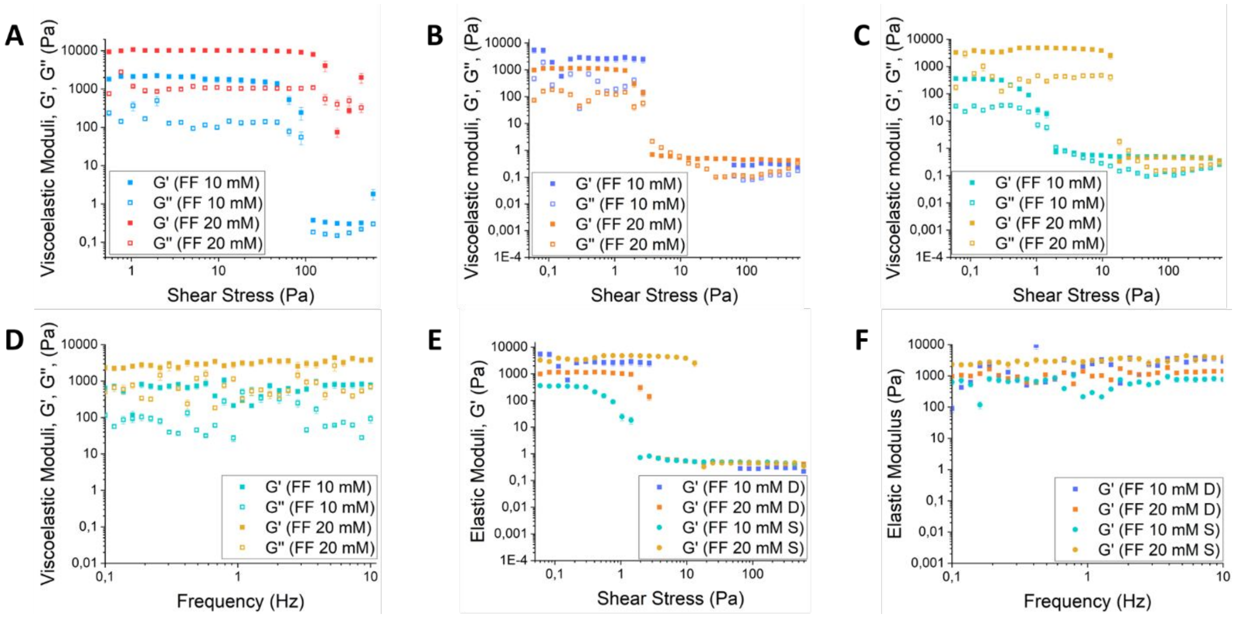

Figure S1. A) Evolution of viscoelastic moduli as a function of shear stress at a constant frequency of 1 Hz of Fmoc-FF hydrogels (10 mM (blue) and 20 mM (red)) formed by the addition of $Zn(AcO)_2$; B) Evolution of viscoelastic moduli as a function of shear stress at a constant frequency of 1 Hz of ZIF-8 composites formed by diffusion protocol (Fmoc-FF 10 mM and HmIm:Zn ratio of 5:1 (violet) and Fmoc-FF 20 mM and HmIm:Zn ratio of 5:1 (orange); C) Evolution of viscoelastic moduli as a function of shear stress at a constant frequency of 1 Hz of ZIF-8 composites formed by simultaneous protocol (Fmoc-FF 10 mM and HmIm:Zn ratio of 5:1 (cyan) and Fmoc-FF 20 mM and HmIm:Zn ratio of 5:1 (mustard); D) Evolution of viscoelastic moduli as a function of frequency at a constant stress of 0.1 Pa of ZIF-8 composites formed by simultaneous protocol (Fmoc-FF 10 mM and HmIm:Zn ratio of 5:1 (cyan) and Fmoc-FF 20 mM and HmIm:Zn ratio of 5:1 (mustard); E) Comparison of the values of the elastic modulus as a function of shear stress of ZIF-8 composites under a constant frequency of 1 Hz; E) Comparison of the values of the elastic modulus as a function of frequency of ZIF-8 composites under a constant shear stress of 0.1 Pa.

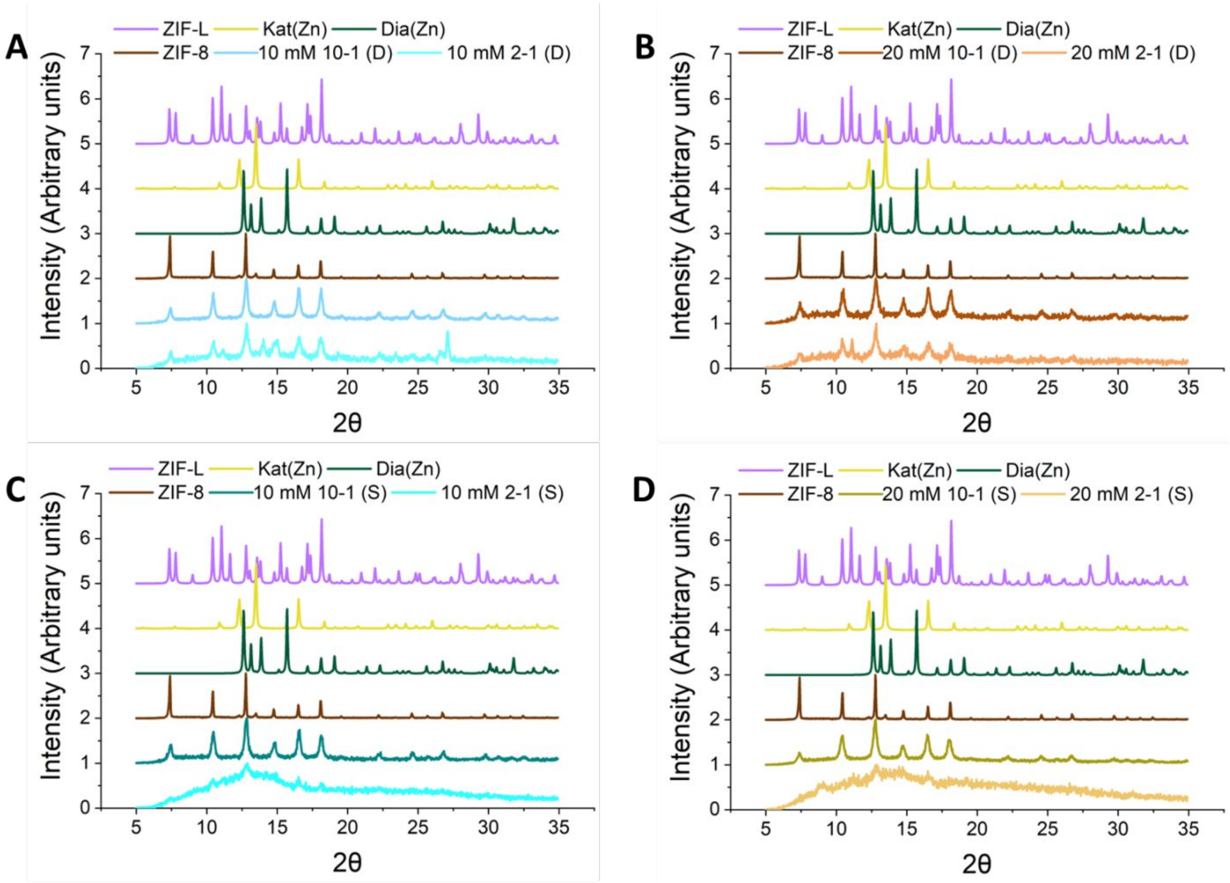

Figure S2. XRPD of ZIF-8 composites obtained in Fmoc-FF hydrogel at different concentrations and protocols compared with the different forms of Zn-MOF obtained in water: in violet ZIF-L, in yellow Kat(Zn), in dark green Dia(Zn), in brown ZIF-8 obtained in water. A) ZIF-8 formed by diffusion protocol (Fmoc-FF 10 mM and HmIm:Zn ratio of 10:1 (light blue) and Fmoc-FF 10 mM and HmIm:Zn ratio of 2:1 (cyan)); B) ZIF-8 formed by diffusion protocol (Fmoc-FF 20 mM and HmIm:Zn ratio of 10:1 (dark orange) and Fmoc-FF 20 mM and HmIm:Zn ratio of 2:1 (light orange)); C) ZIF-8 formed by simultaneous protocol (Fmoc-FF 10 mM and HmIm:Zn ratio of 10:1

(turquoise) and Fmoc-FF 10 mM and HmIm:Zn ratio of 2:1 (cyan)); D) ZIF-8 formed by simultaneous protocol (Fmoc-FF 20 mM and HmIm:Zn ratio of 10:1 (gold) and Fmoc-FF 20 mM and HmIm:Zn ratio of 2:1 (pumpkin)).

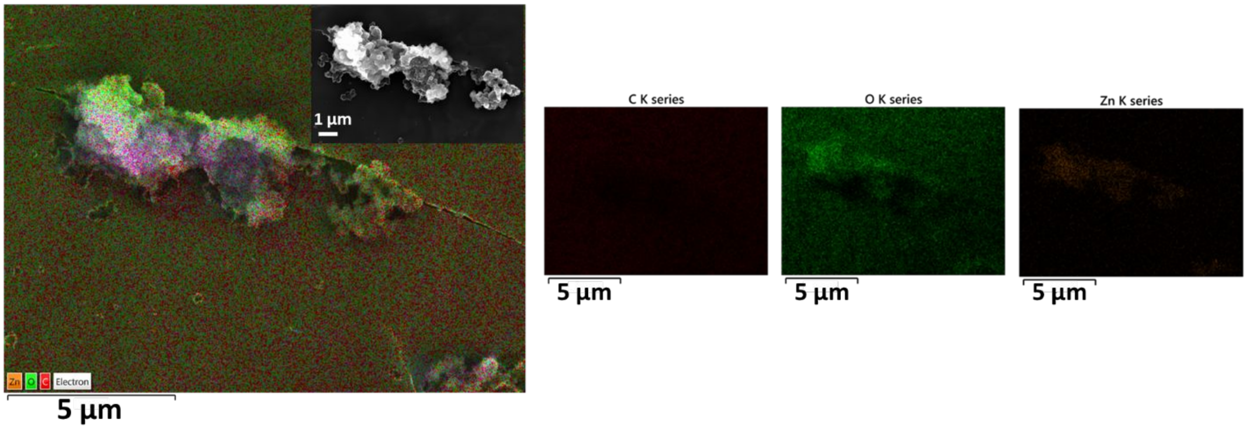

Figure S3. Energy Dispersive Spectroscopy (EDX) analysis of ZIF-8 composites (Fmoc-FF 10 mM, HmIm:Zn ratio of 5:1).

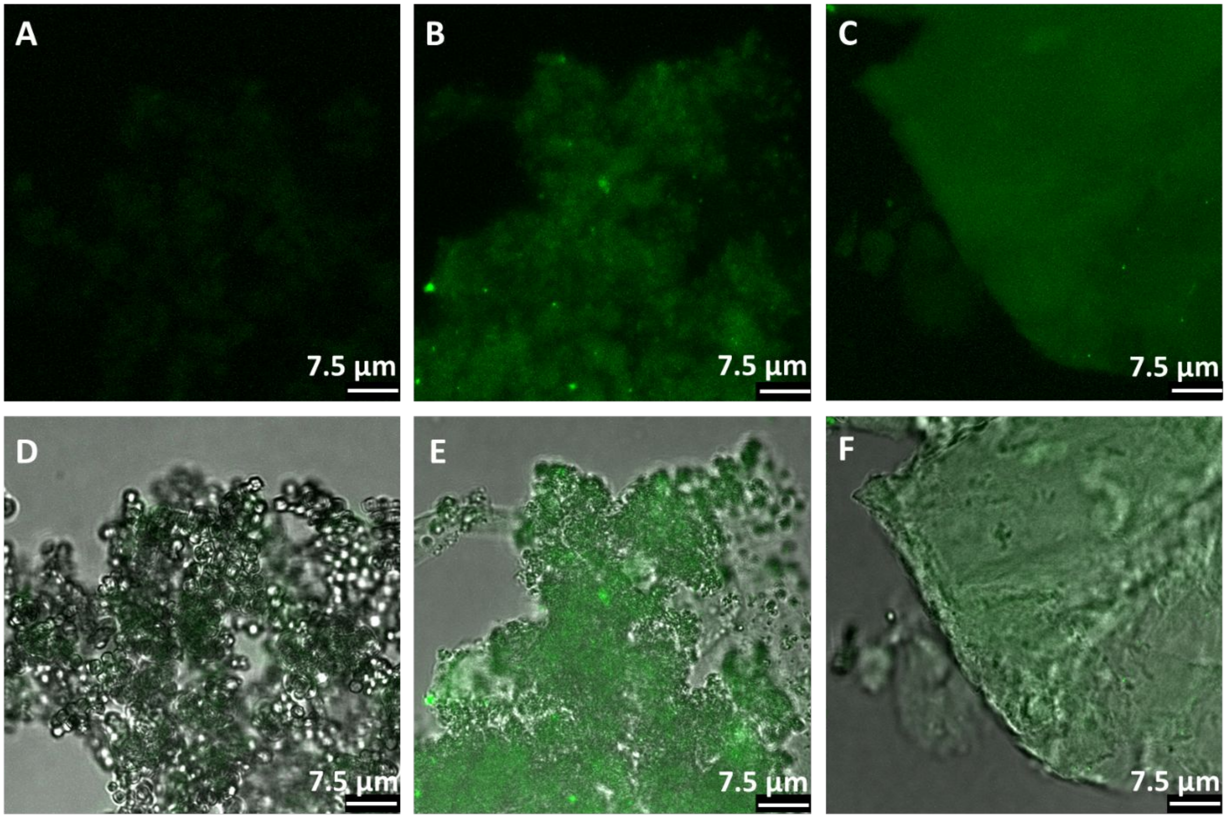

Figure S4. CLSM of A) and D) ZIF-8 grown in water; B) and E) ZIF-8 grown in Fmoc-FF hydrogel formed by diffusion protocol (Fmoc-FF 20 mM and HmIm:Zn ratio of 5:1); C) and F) Fmoc-FF 20 mM hydrogel. A-C) are fluorescence images and D-F) are the overlay with their corresponding visible images.

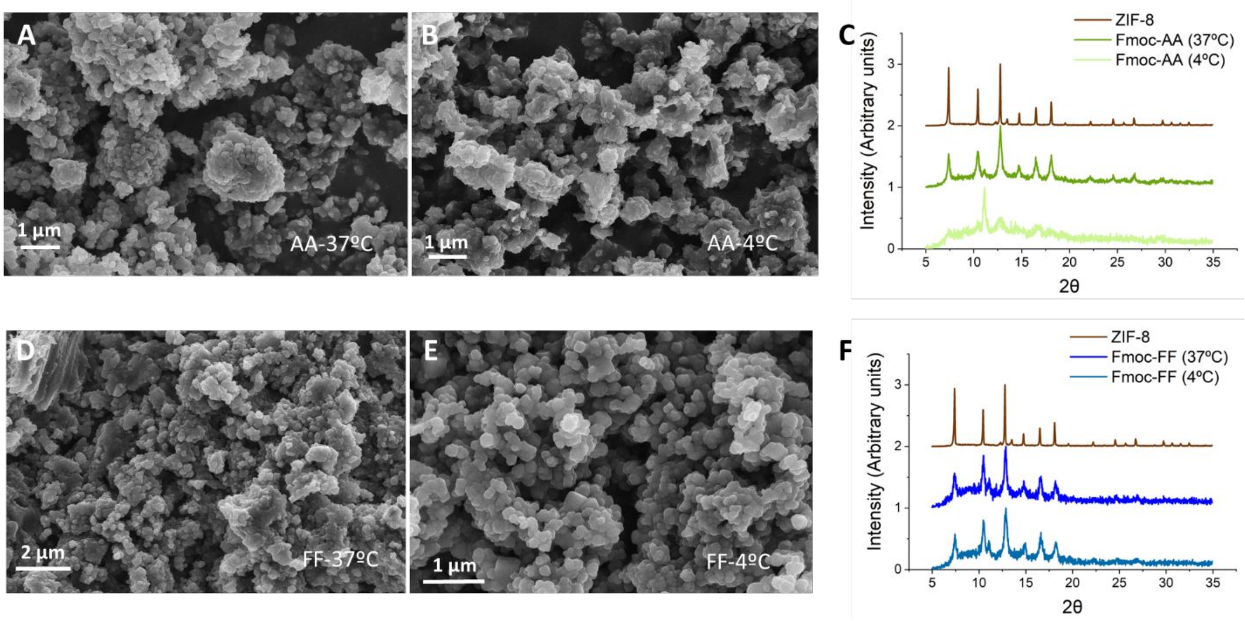

Figure S5. A) SEM image of ZIF-8 composite formed by diffusion protocol at 37 °C (Fmoc-AA 10 mM hydrogel, HmIm:Zn 2:1 ratio); B) SEM image of ZIF-8 composite formed by diffusion protocol at 4 °C (Fmoc-AA 20 mM hydrogel, HmIm:Zn 2:1 ratio); C) XRPD of ZIF-8 composite formed in water (brown), by diffusion protocol in Fmoc-AA hydrogel (10 mM) and HmIm:Zn ratio 2:1 at 37 °C (dark green), by diffusion protocol in Fmoc-AA hydrogel (10 mM) and HmIm:Zn ratio of 2:1 at 4 °C (light green); D) SEM image of ZIF-8 composite formed by diffusion protocol at 37 °C (Fmoc-FF 20 mM hydrogel, HmIm:Zn 2:1 ratio); E) SEM image of ZIF-8 composite formed by diffusion protocol at 4 °C (Fmoc-FF 20 mM hydrogel, HmIm:Zn 2:1 ratio); F) XRPD of ZIF-8 composite formed in water (brown), by diffusion protocol in Fmoc-FF hydrogel (20 mM) and HmIm:Zn ratio 2:1 at 37 °C (dark blue), by diffusion protocol in Fmoc-FF hydrogel (20 mM) and HmIm:Zn ratio of 2:1 at 4 °C (light blue).

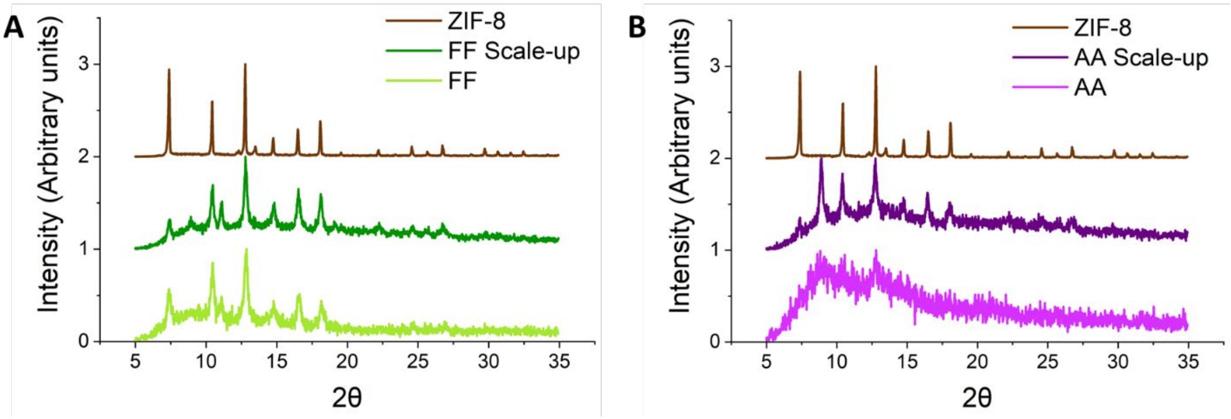

Figure S6. A) XRPD of ZIF-8 obtained in water (brown), by diffusion protocol at 37 ºC (Fmoc-FF 20 mM and HmIm:Zn ratio of 2:1) scale-up (dark green), by diffusion protocol at 37 ºC (Fmoc-FF 20 mM and HmIm:Zn ratio of 2:1) scale-up (light green); B) XRPD of ZIF-8 obtained in water (brown), by diffusion protocol at 37 ºC (Fmoc-AA 10 mM and HmIm:Zn ratio of 2:1) scale-up (dark violet), by diffusion protocol at 37 ºC (Fmoc-AA 10 mM and HmIm:Zn ratio of 2:1) scale-up (light violet).

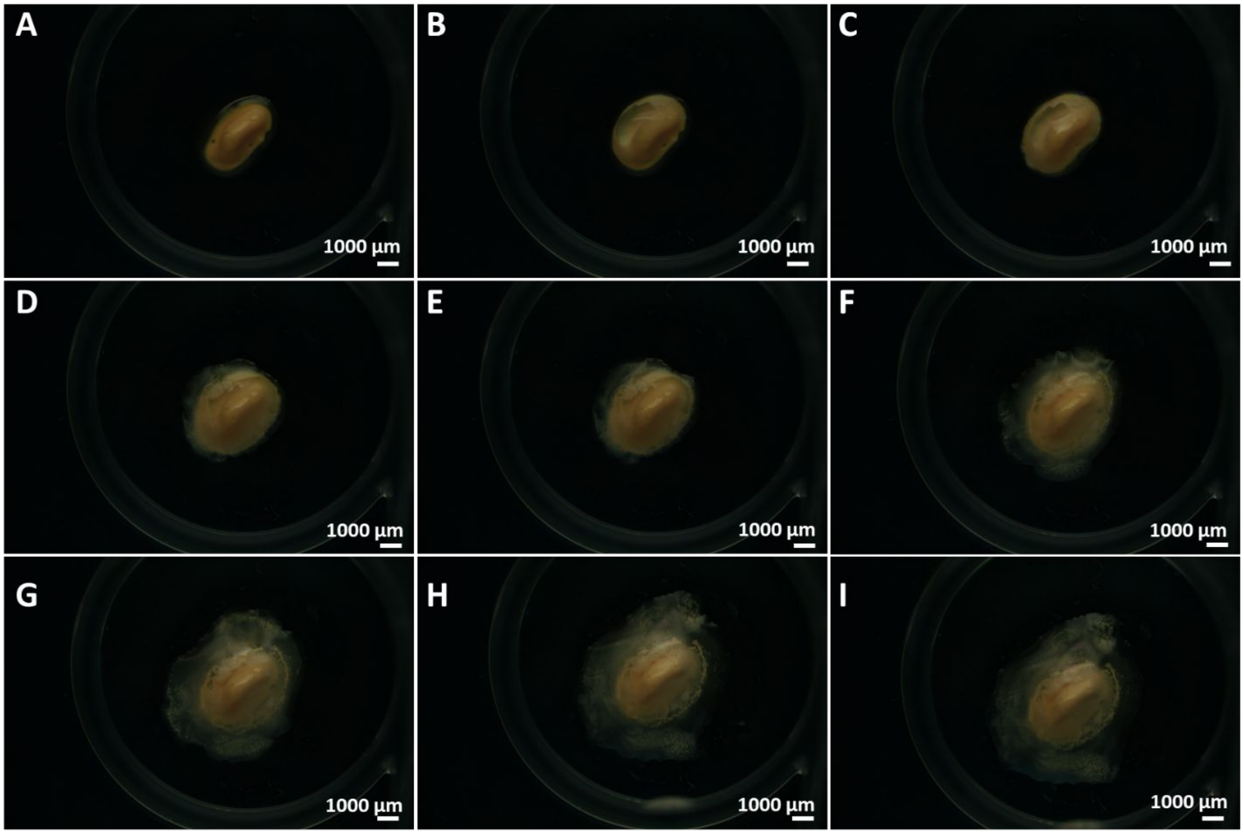

Figure S7. Optical pictures of ZIF-8 composite material: A) after slow-drying (37 ºC 48 h); B) after adding 5 µL of water; C) after 30 s in contact with 5 µL of water; D) after 60 s in contact with 5 µL of water E) after 90 s in contact with 5 µL of water; F) after adding another 5 µL of water (10 µL total); G) after adding another 5 µL of water (15 µL total); H) after adding 15 µL of water (30 µL total); I) after 1 h in contact with 30 µL of water.

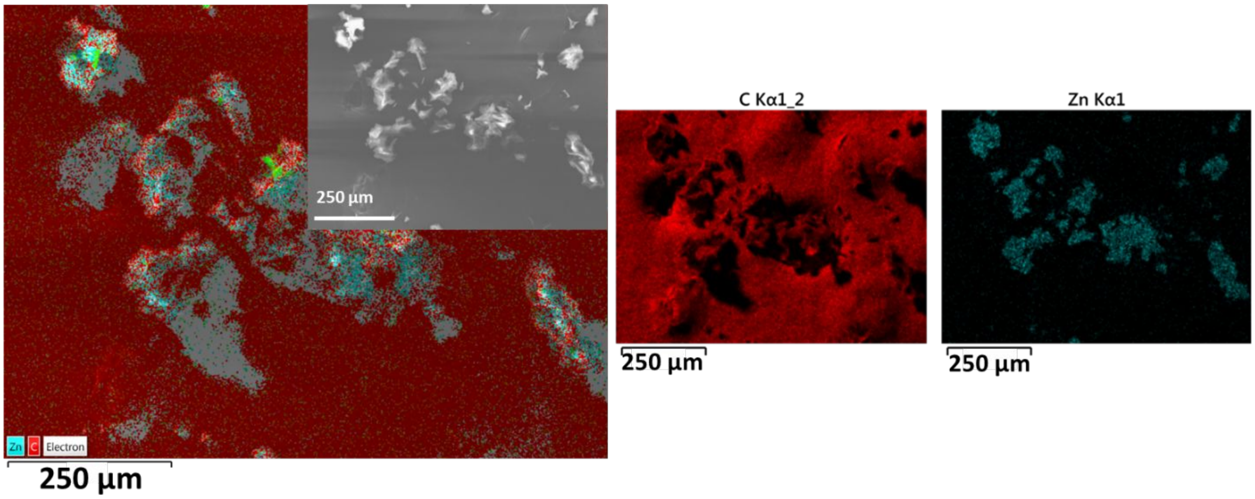

Figure S8. EDX analysis of ZIF-8 composites formed by simultaneous protocol (Fmoc-FF 20 mM and HmIm:Zn ratio of 5:1)

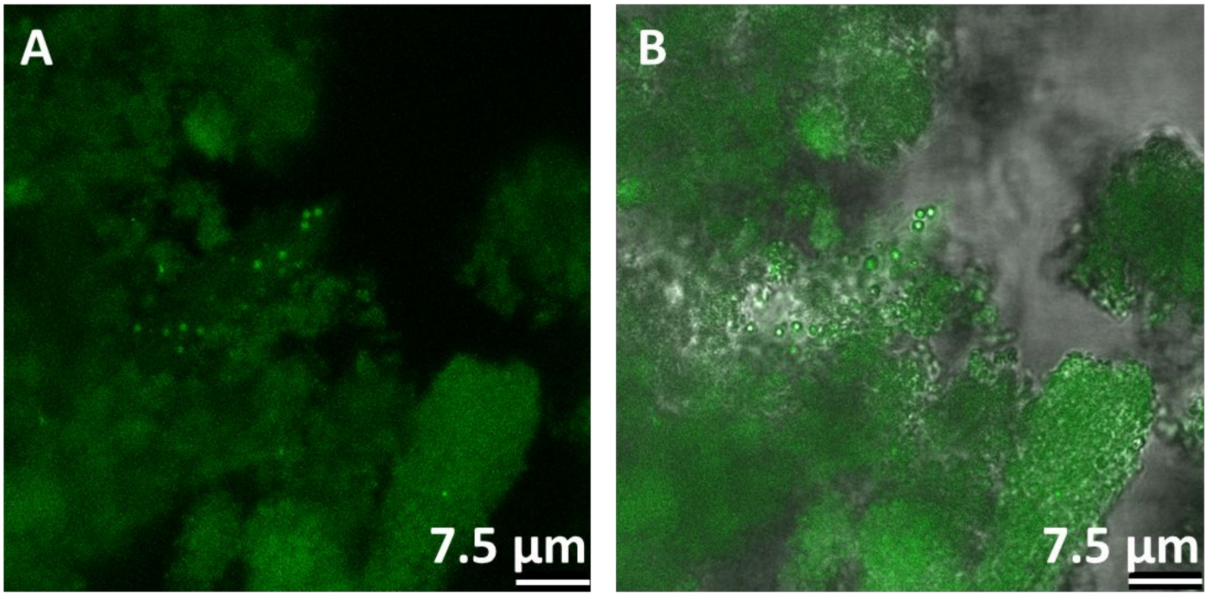

Figure S9. CLSM of ZIF-8 grown by simultaneous protocol (Fmoc-FF 20 mM and HmIm:Zn ratio of 5:1). A) fluorescence image and B) the overlay with their corresponding visible image.

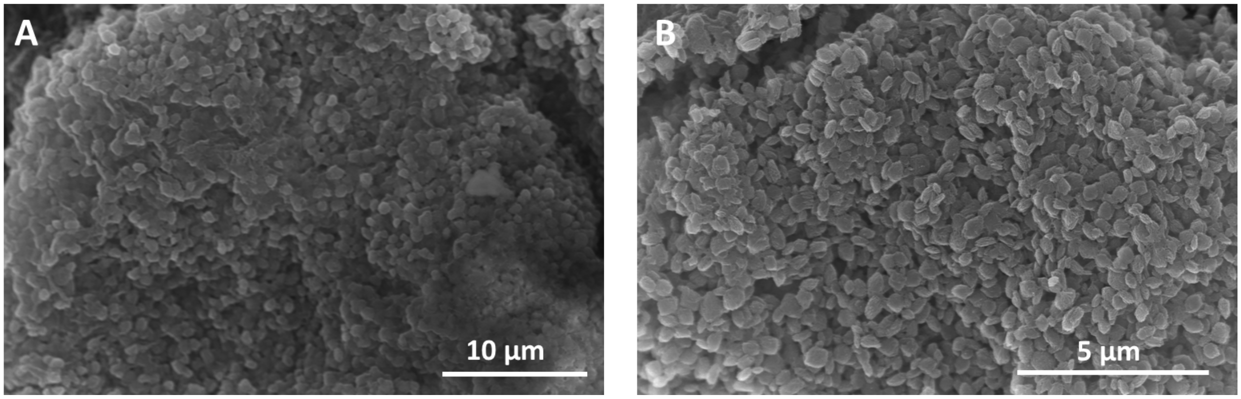

Figure S10. A) SEM image of ZIF-8 obtained by diffusion protocol (Fmoc-FF 20 mM and 10 mM of Zn^{2+}); B) SEM image of ZIF-8 obtained by diffusion protocol (Fmoc-FF 20 mM and 20 mM of Zn^{2+}).

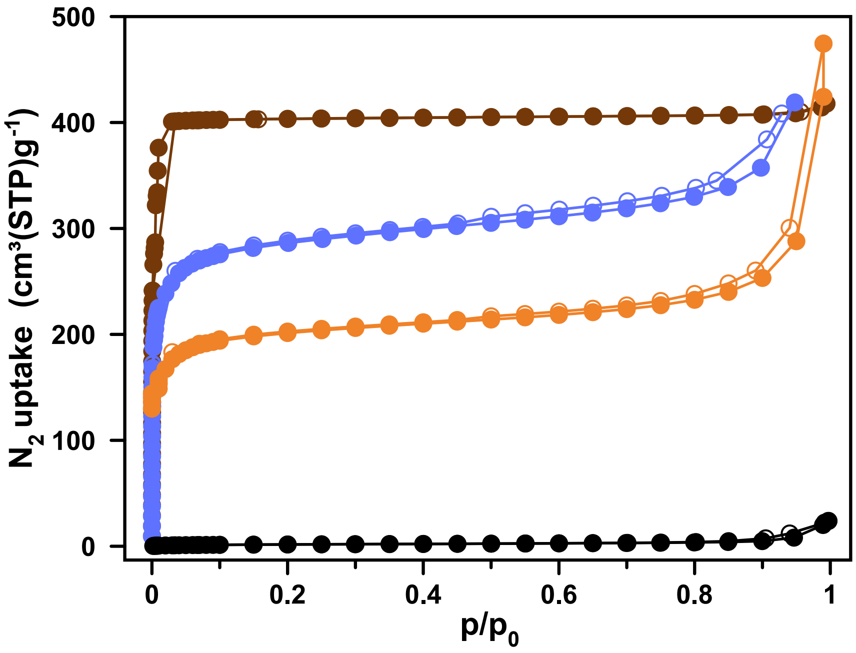

Figure S11. N_2 adsorption isotherms at 77 K of ZIF-8 nanoparticles (brown circles), Fmoc-FF xerogel (black circles) and ZIF-8 composites of Fmoc-FF 10 mM (blue circles) and of Fmoc-FF 20 mM (orange circles).

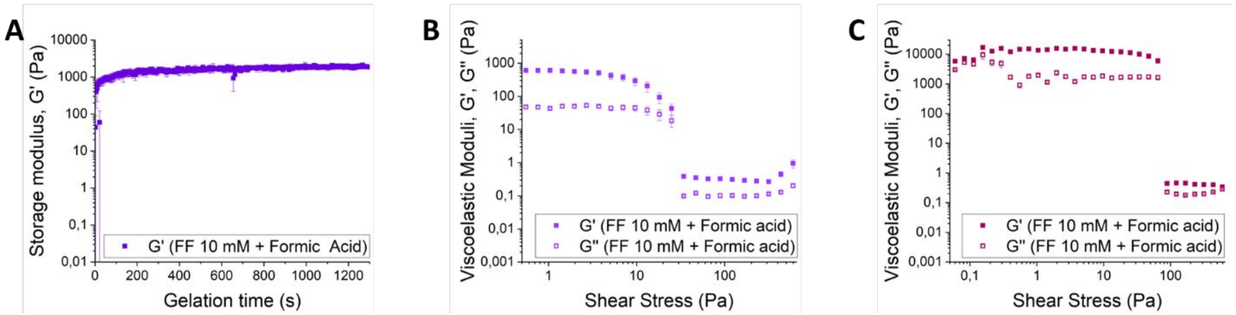

Figure S12. A) Evolution of storage modulus (G') during gelation for Fmoc-FF hydrogels (10 mM) formed with formic acid; B) Evolution of viscoelastic moduli as a function of shear stress of Fmoc-FF hydrogels with formic acid under a constant frequency of 1 Hz; C) Evolution of viscoelastic moduli as a function of shear stress of MOF-808 composite (Fmoc-FF 10 Mm) under a constant frequency of 1 Hz.

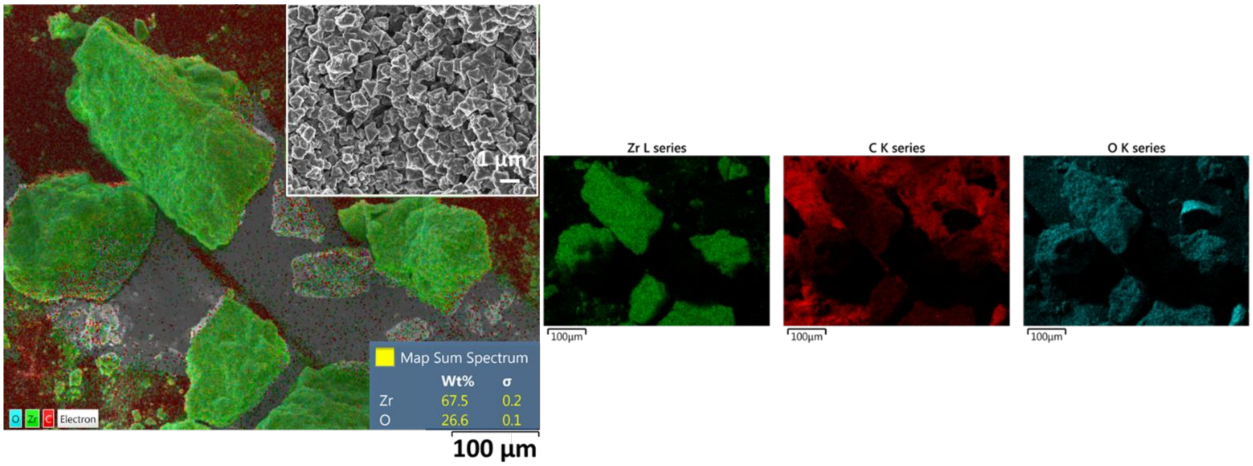

Figure S13. EDX analysis of MOF-808 composites (Fmoc-FF 10 Mm).

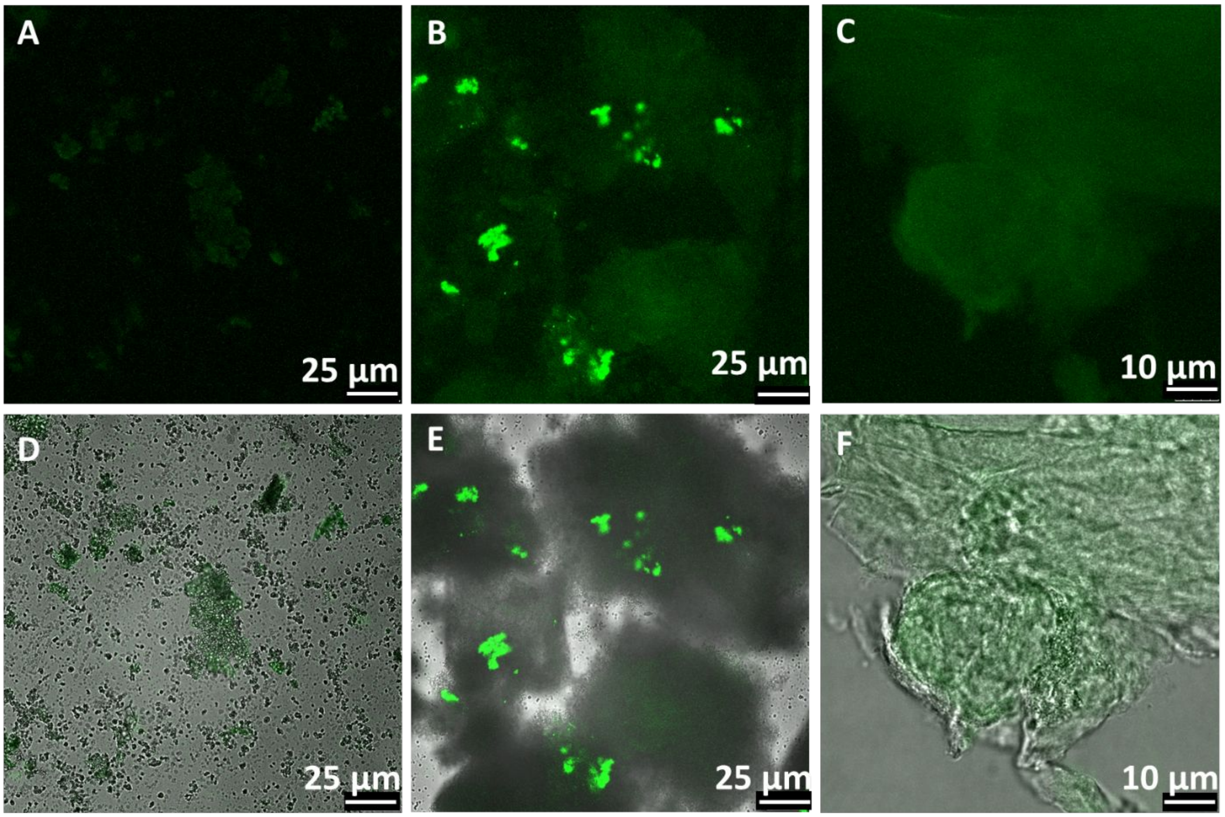

Figure S14. CLSM of A) and D) MOF-808 grown in water; B) and E) MOF-808 grown in Fmoc-FF hydrogel (10 Mm); C) and F) Fmoc-FF 10 mM hydrogel formed with formic acid. A-C) are fluorescence images and D-F) are the overlay with their corresponding visible images.

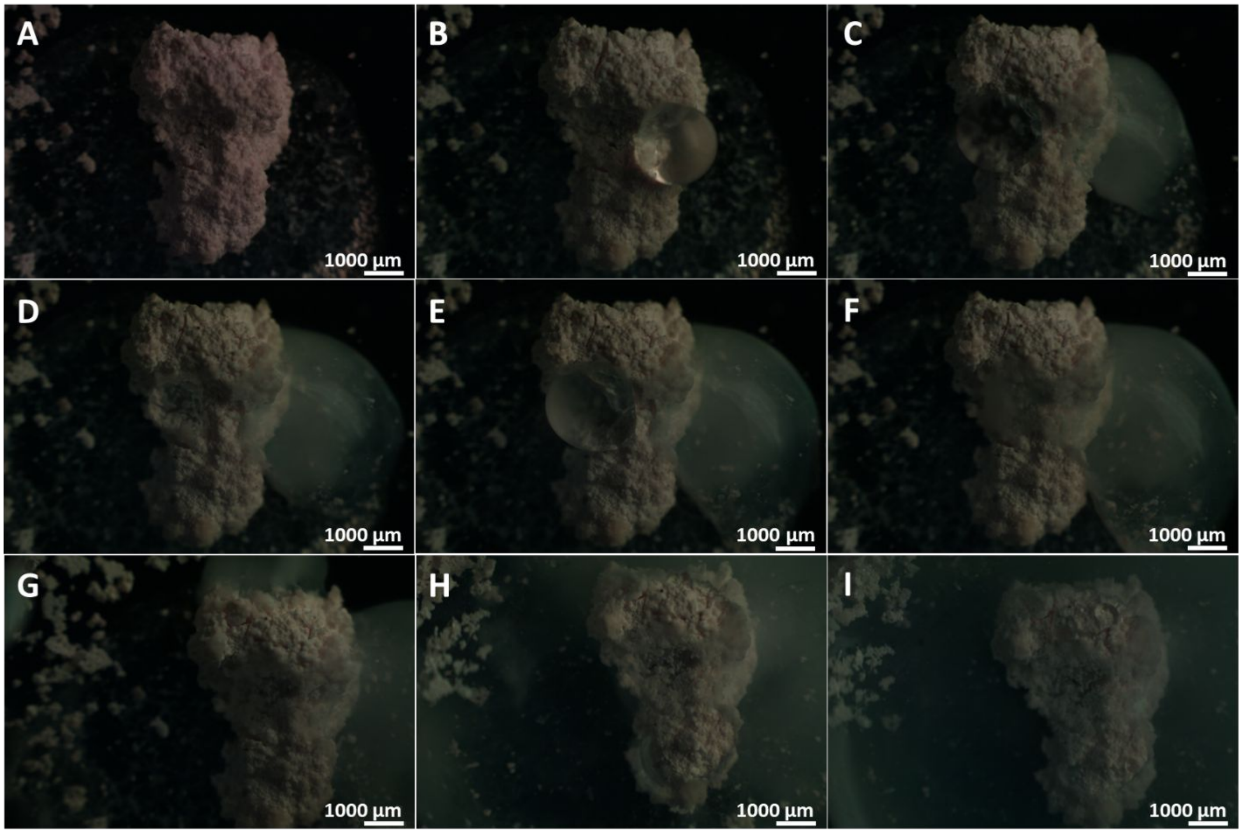

Figure S15. Optical pictures of MOF-808 composite material: A) after slow-drying (37 ºC 48 h); B) after adding 5 µL of water; C) after adding another 5 µL of water (10 µL total); D) after adding 15 µL of water (25 µL total); E) after adding 10 µL of water (35 µL total); F) after adding 20 µL of water (55 µL total); G) after adding 35 µL of water (90 µL total); H) after adding 360 µL of water (450 µL total); I) after 15 h in contact with 450 µL of water.

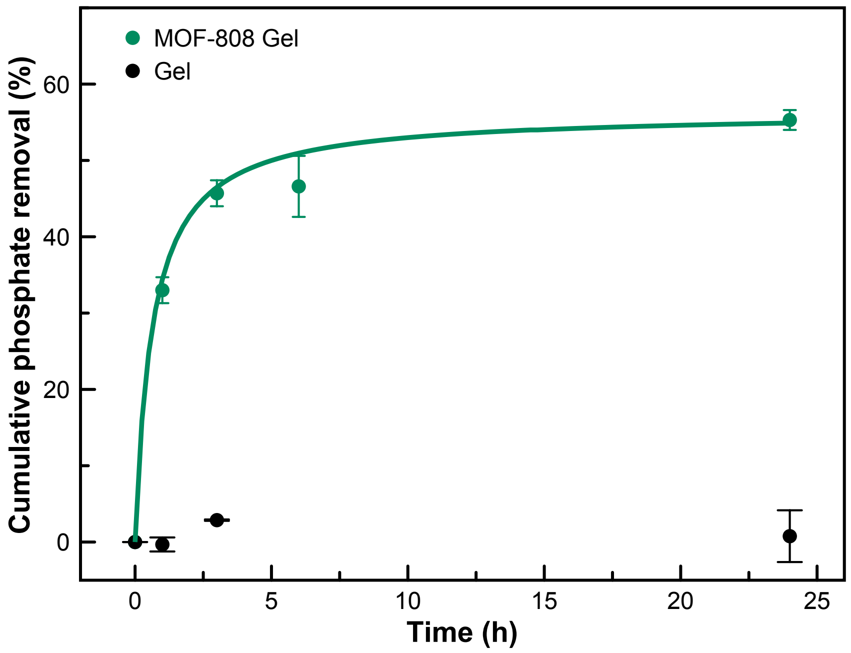

Figure S16. Cumulative phosphate adsorption of MOF-808 peptide hydrogel (green circles) and peptide hydrogel (black circles) at room temperature.

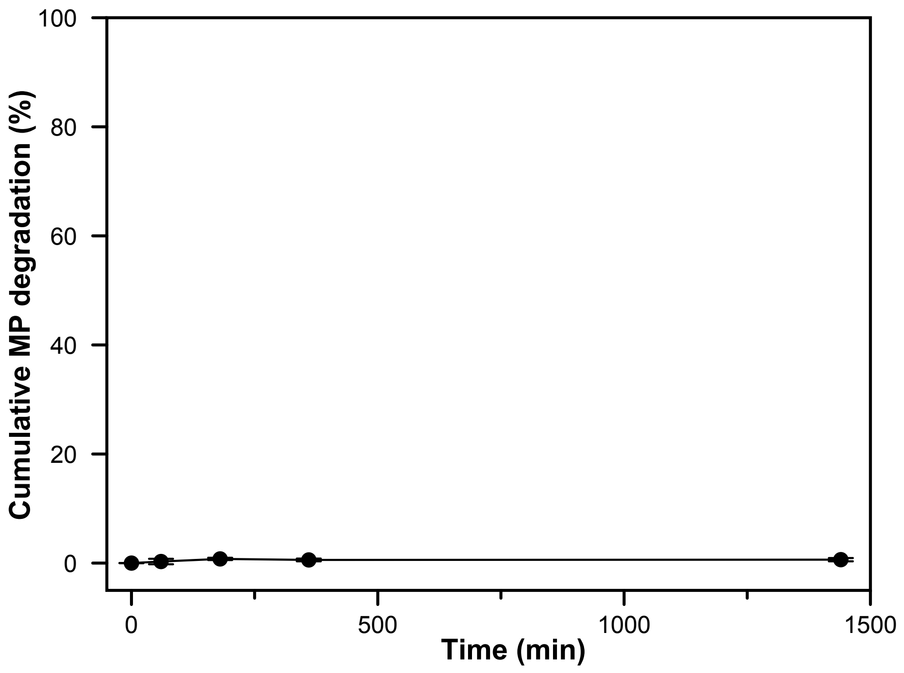

Figure S17. Cumulative degradation of methylparaoxon by dried Fmoc-FF gel (10 mM) at room temperature.

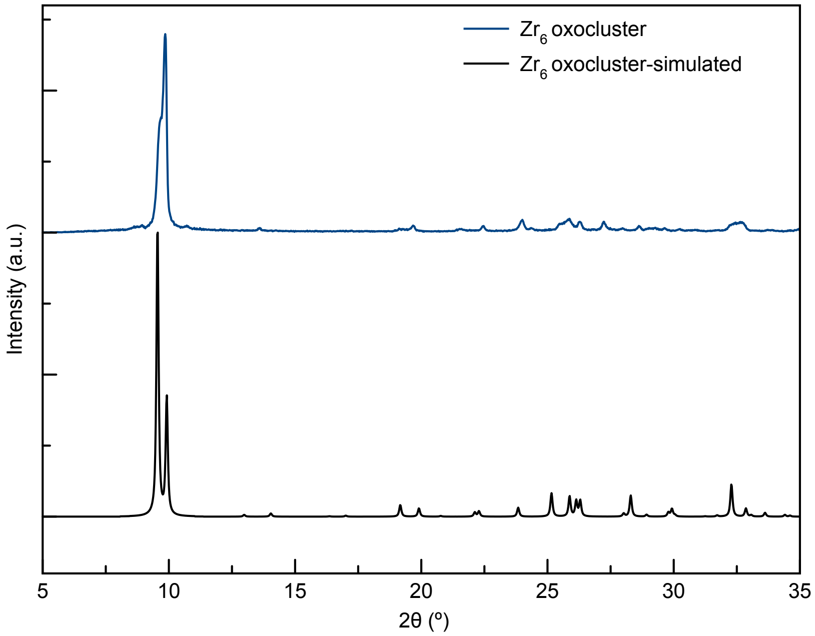

Figure S18. PXRD of Zr_6oxocluster as synthesized (curve line) and simulated (black line).

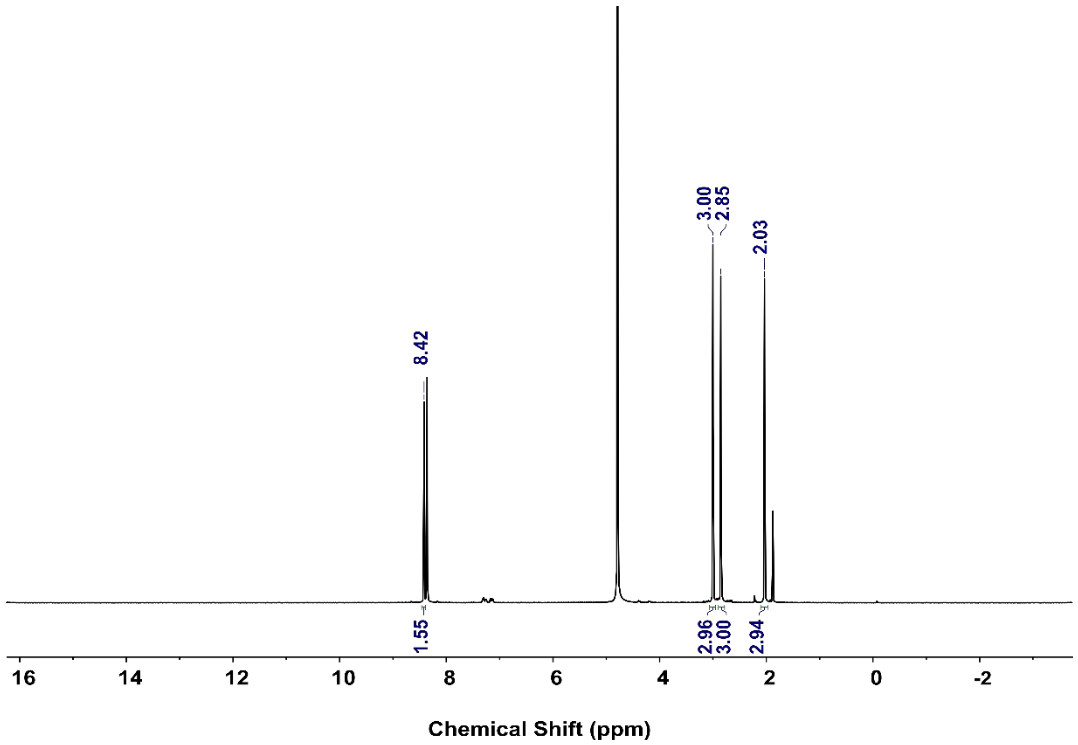

Figure S19. ^{1}H-NMR spectrum of the solution after the digestion of MOF-808-gel (20 mg) with NaOD 1 M during 24 hours at 25 ºC. Internal Reference: Dimethylacetamide (DMA, 0.028 M). The signal at $\delta = 8.42$ ppm is assigned to trimesate ions (3H).
